# Printed Diodes: Materials Processing, Fabrication, and Applications

**DOI:** 10.1002/advs.201801653

**Published:** 2019-01-30

**Authors:** Yihang Chu, Chunqi Qian, Premjeet Chahal, Changyong Cao

**Affiliations:** ^1^ Laboratory for Soft Machines & Electronics School of Packaging Michigan State University East Lansing MI 48824 USA; ^2^ Department of Electrical and Computer Engineering Michigan State University East Lansing MI 48824 USA; ^3^ Department of Radiology Michigan State University East Lansing MI 48824 USA; ^4^ Department of Mechanical Engineering Michigan State University East Lansing MI 48824 USA

**Keywords:** nanomaterials, organic light‐emitting diodes (OLEDs), printed diodes, printing technologies, radio frequency identifications (RFIDs)

## Abstract

Printing techniques for the fabrication of diodes have received increasing attention over the last decade due to their great potential as alternatives for high‐throughput and cost‐effective manufacturing approaches compatible with both flexible and rigid substrates. Here, the progress achieved and the challenges faced in the fabrication of printed diodes are discussed and highlighted, with a focus on the materials of significance (silicon, metal oxides, nanomaterials, and organics), the techniques utilized for ink deposition (gravure printing, screen printing, inkjet printing, aerosol jet printing, etc.), and the process through which the printed layers of diode are sintered after printing. Special attention is also given to the device applications within which the printed diodes have been successfully incorporated, particularly in the fields of rectification, light emission, energy harvesting, and displays. Considering the unmatched production scalability of printed diodes and their intrinsic suitability for flexible and wearable applications, significant improvement in performance and intensive research in development and applications of the printed diodes will continuously progress in the future.

## 1 Introduction

As one of the most ubiquitous electronic components, diodes have been intensively studied over the past century, driven by both commercial demand as well as potential for improvement.^1^, ^2^, ^3^, ^4^ The widely utilized ability of diodes to control the unidirectional flow of current originates from a structural junction permitting the flow of current in one direction, but not the other.^5^, ^6^ Their usage in voltage regulation and voltage surge protection pushed development toward alteration of the reverse breakdown voltage, while the discovery of light‐emitting diodes (LEDs) led to the opening of an entire dedicated subfield of research in response to the tremendous commercial potential. Amid these influences, the meteoric rise of rectifiers relying on diodes and their use in near‐field communication and wireless power transmission technology gave rise to a comprehensive push toward diodes of higher switching speeds capable of radio frequency (RF) operation. In the 1970s, an associated surge of interest in the development of RF identification (RFID) tags intensified the demand for flexible electronic components. This trend, when combined with the ever‐ongoing search for less costly methods of mass production, inevitably propelled research on diodes to the field of printed electronics.

Among diode fabrication methods, printing is an umbrella term incorporating all techniques in which solution‐processed materials are deposited in the form of ink on a substrate. Printed diode designs generally attempt to minimize the number of layers due to the difficulty of multilayer printing originating from limitations of solvent processing, and typically are based on vertical structures to reduce demands on lateral printing resolution.^7^ The processing methods of this category generally excel in the scalability, cost‐effectiveness, efficiency, and production speed that are vital to the widespread use of dependent technologies such as RFID tags or flexible LEDs.^1^, ^8^ Another vital advantage stems from the compatibility of printing with thin, flexible, and lightweight substrate materials, except in cases such as metal oxide semiconductors in which the solution‐processed materials require high temperature annealing which may damage substrates with lower melting points.^9^ For ultrahigh frequency (UHF) applications, printing manufacturing can also potentially bypass component assembly steps by directly printing preconnected components.^10^, ^11^ Despite such overwhelming advantages, certain drawbacks of printing techniques have limited their use. For instance, the requirement of solution‐processing of needed materials can compromise their electrical properties, especially certain sensitive materials, resulting in lowered device performance. Also, the formation of excellent metal–semiconductor contacts at room temperature remains a major challenge. Additionally, due to the difficulty of multilayer printing, a large portion of printed diodes are single layer unipolar devices based on a single semiconductor of either electron or hole conduction properties.

**Figure 1:**
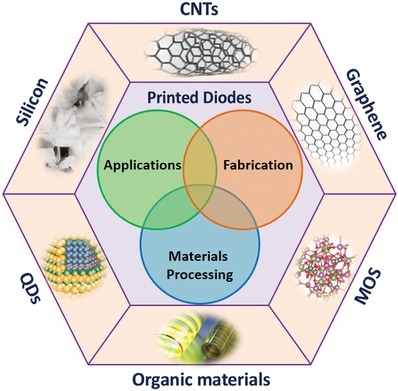
Outline illustration of the review for printed diodes via different kinds of materials. The focus of the review is placed on materials processing, fabrication approaches, and broad applications of the diodes and their integrated devices.

In this review, we summarize the progress of recent advances regarding the material processing, fabrication, and applications of printed diodes, with an emphasis placed on the aspects of solution‐processing and material performance (**Figure** **1**). A brief overview for printed diodes is first presented in Section 2 describing the general architectures used in printed diodes, as well as the criteria through which they are evaluated. Section 3 summaries the major printing processes for fabricating printed diodes. Afterward, the subsequent Section 4 focuses on the primary classes of materials used in printing diodes, with each subsection highlighting the notable advances, detailing challenges, and summarizing their applications. In detail, Section 4.1 is devoted to silicon, the dominant material within modern rigid‐substrate semiconductor technology; Section 4.2 focuses on metal oxide semiconductors (MOS), a class of materials which has progressed greatly over the last decade; Section 4.3 describes a variety of nanomaterials, which have demonstrated the greatest electrical performance and potential, but also must overcome a few grand challenges for commercially viable applications; Section 4.4 discusses the organic materials widely used in printed diodes, which possess the greatest compatibility with printing fabrication methods, but suffer from a lack of high performance and device longevity due to their limited material properties. Finally, a brief conclusion and discussion for the future of printed diodes is presented in Section 5.

## 2 Types and Frequency of Diodes

### 2.1 Schottky Diode

In its simple form, the Schottky diode consists of one layer of a p‐type or n‐type semiconductor positioned between two metal electrode contacts, one of which permitting charge injection in one bias direction while blocking in the other, and the other providing low resistance current conduction as an ohmic contact. Where the rectifying metal electrode and the semiconductor are brought together, the originally different Fermi level of the semiconductor and work function of the metal are forced to align, theoretically resulting in band bending and the creation of the Schottky barrier responsible for rectification as described by the Schottky–Mott rule.^8^ In practical applications, however, the Schottky–Mott rule is not strictly followed by metal–semiconductor interfaces due to the phenomenon of Fermi level pinning, which originates from the formation of electron states within the bandgap at the termination of the semiconductor material against the rectifying metal contact.^12^

In the unipolar Schottky diode, there is no charge carrier depletion region at the metal–semiconductor junction. Thus, under forward bias, holes can shift without much hinderance from the semiconductor directly into the metal, while the Schottky barrier blocks hole injection from the metal in the reverse direction. The lack of a charge carrier depletion region allows for the elimination of the reverse recovery time required in other types of diodes for shifting operation state from forward bias to reverse bias, resulting in the outstanding switching frequency of Schottky diodes. Additionally, as the metal–semiconductor barrier is lower in height compared to the junction barriers in p–n junction diodes, a lower activation voltage in forward bias is required, while yielding a higher forward current density and lower forward bias voltage drop.^1^ In return, however, the low barrier height is a disadvantage in reverse bias, being responsible for the higher levels of reverse leakage current and reduced reverse breakdown voltage which have limited the Schottky diode to low‐voltage applications. These two disadvantages can be compensated for architecturally by surrounding the diode with semiconductor guard ring structures, which can guard against leakage current and reverse breakdown by controlling the breakdown field geometry.^13^ The Schottky diode has emerged as the most popular printed diode design, being well ahead of most other competitors. This diode architecture has demonstrated both excellent printing compatibility and outstanding switching frequency performance at lower voltages.

### 2.2 Tunnel Diodes

Tunnel diodes have shown the potential for impact in low‐energy applications emphasizing durability, in which negative differential states can be observed when quantum mechanical tunneling occurs through a thin barrier.^14^ The initial design for tunnel diodes took advantage of this with a modified p–n junction architecture in which the valence band hole states of the p‐type semiconductor were nearly aligned with the conduction band electron states of the n‐type semiconductor, either through heavy doping or through employing the natural electrical properties of the chosen materials. Moreover, the tunneling mechanism has also been observed and applied to notable effect in metal–oxide–metal and metal–insulator–metal (MIM) diode architectures, which rely on the work function difference between the narrowly separated metal layers to generate relatively asymmetric *I*–*V* characteristics.^15^

As the speed of quantum tunneling far exceeds the speed of conventional drift currents or diffusion currents which rely on conduction, tunnel diodes have the potential for unsurpassed high frequency operation under extremely low bias voltages.^8^, ^16^, ^17^ Tunnel diodes have also demonstrated excellent durability and longevity as well as superb resistance to ionizing radiation, which allow them to be used in space and other high‐radiation environments.^18^ In addition, MIM tunnel diodes have also been considered for incorporation in image display technology such as liquid crystal display screens as switching devices with reduced cost in comparison to thin‐film transistor (TFT)‐based alternatives.^19^ The use of the negative differential resistance effect for tunnel diodes in printed electronics currently remains at the early stage, although multiple hybrid organic–inorganic tunnel diode architectures with potential for printing fabrication have been reported.^8^, ^20^

### 2.3 LEDs

Organic LEDs (OLEDs) are dominating the current printed LEDs field due to their superior contrast ratio, response time, and power efficiency, as well as the unmatched compatibility with solution‐processing approaches of cheap and lightweight organic materials. The most basic single‐layered OLED consists of an emissive layer (EML) of either fluorescent or phosphorescent organic material sandwiched between two electrodes, the anode being transparent and the cathode being reflective or vice versa.^21^ Once voltage is applied, holes and electrons are injected into the organic material layer to form excitons, which become de‐excited after the emission of a photon through the transparent electrode. A more efficient architecture requires the addition of a hole‐transport layer and an electron‐transport layer sandwiching the EML, and further tuning of emitted light properties such as color or intensity may require the addition of supplementary emission layers.^22^, ^23^ To combat the short lifespan of organic materials while preserving the light intensity level, the operation current is designed to be reduced by stacking two or more OLEDs with a charge generation layer in between to double the number of layers,^7^ which has inevitably rendered the advancement of multilayer printing fabrication, a fiercely pursued research topic.^21^, ^24^

Traditional LEDs formed from inorganic materials usually have longer lifespans and adequate energy efficiency.^25^ However, due to the fact that inorganic materials are not compatible with solution‐processing, research on printing inorganic LEDs has received less fervor than that of OLEDs. Recently, quantum dots (QDs) have emerged as another highly suitable material category for printed LEDs.^26^ QD‐LEDs possess many of the advantages of OLEDs such as high efficiency, luminosity, flexibility, and a greater spectrum of visible light, while overcoming lifespan challenges.^27^

The fabrication of LEDs represents a unique challenge to the printed electronics industry. Different types of materials, including organic polymers, organic small molecules, colloidally synthesized quantum dots, and some inorganic materials, have been explored for boosting the performance of printed LEDs. However, regardless of material types, the device architectures of LEDs rely heavily on multiple layers. With the limitations of solvents, multilayer printing faces difficulties in aligning different printed layers to the required degree of accuracy. This shortcoming typically results in devices of lower power efficiency and greater fragility, a phenomenon which has greatly hindered the large‐scale commercialization of LEDs fabricated through printing.^3^, ^7^, ^28^

### 2.4 Operation Frequency

Operation frequency is one of the most important parameters for evaluating the performance of diodes and for determining their applications in different fields such as rectification, signal modulation, and flexible electronics.^2^, ^29^ High frequency (HF) operation (3–30 MHz) and UHF operation (>30 MHz) have been explored intensively in the near‐field communication and energy transmission/harvesting industries.^1^ Being a vital variable of RFID systems, the diode switching frequency, i.e., the rate at which diodes can shift between forward and reverse bias, typically becomes the limiting factor barring rectifiers from higher operation frequencies.^1^, ^30^

**Figure 2:**
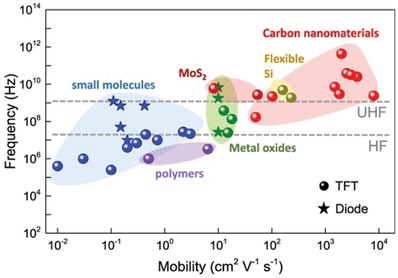
Variation of device frequency characteristics and charge carrier mobility for different kinds of semiconducting materials. Reproduced with permission.^1^ Copyright 2017, IOP Publishing.

Multiple parameters determine the switching frequency of diodes, mainly stemming from the characteristics of materials used as well as the diode structure chosen. First, the two primary material‐dependent parameters are the charge carrier mobility, determining how quickly charge carriers can move through the material under the influence of an electric field, and charge carrier concentration, referring to the concentration of charge carriers within the material per unit of volume. As seen in **Figure** **2**, the different materials possess an array of charge carrier mobility ranges which determine and limit their maximum potential switching frequencies when used in diodes, with carbon nanotubes (CNTs) and graphene demonstrating the highest value range, and organic polymers displaying the lowest.^8^, ^31^ In the research involving the solution‐processing of semiconductor materials, much effort is placed on the preservation of high charge carrier mobility through improving the fabrication process, in particular for organic materials.^32^ On the other hand, charge carrier concentration depends less on the natural performance of the material, and can be artificially enhanced during the fabrication process through doping with many inorganic semiconductor materials.^8^, ^28^

Alongside material considerations, the diode structures chosen are also critical in determining their frequency performance. The most vital parameter dependent on diode structure is the reverse recovery time, which represents the time required for the diode to shift from the conducting to the nonconducting state following a voltage shift. For p–n junction diodes, a charge carrier depletion region forms at the junction under bias, and must require a reverse recovery time ranging from several microseconds to less than 100 ns to disappear before switching.^33^ By contrast, Schottky diodes do not possess a charge carrier depletion region, and thus do not require any reverse recovery time. This advantage is instrumental in establishing the superiority of Schottky diodes in high frequency operation. Another parameter is forward voltage drop, which represents the minimum forward bias voltage at which the diode begins to conduct.^34^ Lower forward voltage drop brings a positive influence on operation frequency, while also leading to higher reverse leakage current and lower reverse breakdown voltages under reverse bias, which may become an issue to the relevant diode architectures, especially for Schottky diodes.

A key measure of diode frequency of operation is the cut‐off frequency, above which the nonlinear characteristics of the diode are significantly suppressed due to the associated parasitics (resistance, capacitance, and inductance).^17^, ^35^ To maximize the cut‐off frequency, the effective series resistance which may arise from poor semiconductor–electrode contact as well as resistivity of the metal and the semiconductor should be kept as low as possible (ideally <1 Ω). In this regard, the fabrication of electrodes becomes of particular importance, because the quality of the semiconductor–electrode junction alongside the associated resistance may greatly affect the device performance. Ideally, diodes with low turn‐on voltage, high cut‐off frequency, and low leakage are desired for RFID applications where power dissipation is a critical design challenge. Also requiring minimization is the diode capacitance, which largely manifests due to the depletion region and the spacing between the metal electrodes.

In the efforts to reduce the capacitance and effective resistance, two diode architectures have risen to prominence. The vertical sandwich‐like diode architecture, consisting of neatly stacked layers, emphasizes the minimization of layer thickness to reduce the effective resistance, but it shows a diode capacitance that scales with diode area, thus limiting the area‐dependent current driving capabilities in return for excellent frequency performance.^36^ For some applications in which the effective impedance of the diodes must be tailored for ease of impedance matching to an external circuit or antennas, the manipulation of diode area for the vertical diode architecture represents an easier and more realistic route. On the other hand, the coplanar diode architecture is formed by depositing semiconductor material in the gap between two electrodes, using the interelectrode distance to define the semiconductor channel length.^37^ Once fabricated, the coplanar diode architecture exhibits ultralow effective resistance and capacitance values which generally result in superior operating frequencies and lower turn‐on voltages. However, the implementation of this structure, especially for Schottky diodes is extremely challenging due to the need for fabricating two asymmetric electrodes within 10–20 nm of distance. This requirement has barred such architecture from any form of large‐scale use even employing the adhesion lithography technique.^36^, ^37^, ^38^, ^39^

## 3 Printing Techniques for Diodes

Among viable fabrication techniques for electronics, printing approaches have received significant attention from both academia and industry due to their ability to bypass the mainstream rigid and expensive silicon‐based electronics to directly deposit preconnected device structures on flexible substrates in an efficient, scalable, and cost‐effective manner.^1^, ^8^, ^9^, ^27^ There are two primary challenges within the process of printing, i.e., the solution‐processing of electronics materials into inks for selected printing fabrication methods^1^, ^2^, ^27^ and the fabrication of multilayer structures with high resolution, good alignment, and favorable compatibility between layers.^7^, ^21^, ^40^, ^41^

**Figure 3:**
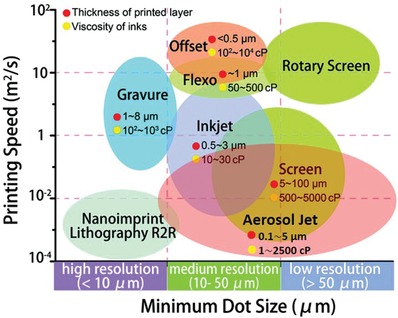
Phase‐diagram of the printing resolutions and speeds demonstrated by the major printing techniques. For each method, the minimum layer thickness and required ink viscosity are presented. Reproduced with permission.^27^ Copyright 2017, Royal Society of Chemistry.

Printing fabrication methods can be generally categorized into sheet‐based and roll‐by‐roll‐based approaches.^42^ For high volume fabrication emphasizing scalability, the roll‐to‐roll (R2R) techniques of gravure, flexographic, and rotary screen printing are ideal. However, when superior printing resolution is required, inkjet printing, screen printing, aerosol jet printing, and gravure printing stand out. A comparison between these printing techniques is summarized in **Figure** **3**, including the printing resolution, printing speed, minimum layer thickness, and required ink properties. We briefly discuss the popular printing approaches used in fabricating printed diodes as below in this section.

### 3.1 Inkjet Printing

**Figure 4:**
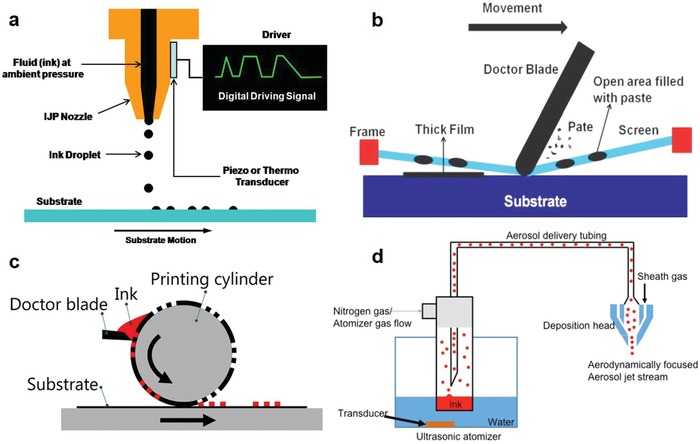
Schematic illustration of the most popular printing techniques. a) Inkjet printing. b) Screen printing. Reproduced with permission.^27^ Copyright 2017, Royal Society of Chemistry. c) Gravure printing. Reproduced with permission.^42^ Copyright 2016, IOP Publishing. d) Aerosol jet printing. Reproduced with permission.^191^ Copyright 2017, IOP Publishing.

As illustrated in **Figure** **4**a, inkjet printing relies on the ejection of droplets of ink from a nozzle onto the substrate, which can be either rigid or flexible. Two different methods of drop‐on‐demand ink ejection exist: 1) the piezoelectric method, in which a piezoelectric ceramic tile is used to generate pressure to force drops of ink from a reservoir near the nozzle, and 2) the thermal method, in which thermal excitation from a heating element is used to cause rapid vaporization of the ink to form a bubble, resulting in a rapid pressure increase capable of propelling a droplet of ink out through the nozzle.^27^, ^43^, ^44^ Typically, the inks for this technique are formulated with viscosities on the order of 1–20 centipoise, similar to that of water, to maintain good jettability of the inks. As a noncontact printing method, inkjet printing can perform moderately detailed patterning without the use of any mask. Compared to most other printing techniques, inkjet printing possesses one of the lowest percentages of wasted materials during fabrication as well as low initial startup costs, thus rendering it the easiest printing tool to employ when convenience is the first priority.^45^ However, due to the limitation of the printing technique, inkjet printers cannot handle highly viscous inks and high aspect ratio particle inks, such as organic dielectrics and CNTs, which may lead to severe nozzle clogging.^43^, ^46^, ^47^ Though excelling in neither printing resolution nor scalability, inkjet printing presents a balance between these parameters, a technique most suitable for industrial production, definitively surpassed only by gravure printing.^43^, ^44^ Inkjet printing has been widely used for printing conductive electrodes with nanoparticle inks.^48^

### 3.2 Screen Printing

Screen printing is a technique in which a fine patterned mesh of either porous nylon textile or metal stretching over a rigid frame is used to deliver ink on a substrate, as seen in Figure 4b.^27^ The screen is placed above a substrate and ink is applied to it. With the assistance of a squeegee, the mesh is pressed to the substrate along a line of contact, allowing ink to be transferred to the substrate in accordance to the patterns of the mesh along the mesh openings.^49^ Despite seeing less attention than inkjet printing, screen printing is noted for the capability to produce thick, patterned layers from materials of high viscosity while maintaining throughput and resolution of approximately the same order of magnitude as inkjet printing.^50^ The greatest strength of screen printing is its versatility, applicable for solution‐processed inorganic and organic materials of all viscosities regardless of layer function or substrate flexibility.

### 3.3 Gravure Printing

Gravure printing in particular has received intensive attention due to its excellent scalability and competitive resolution compared with inkjet and screen printing.^27^ Employing a printing process like offset printing and flexography, gravure printing requires the desired image be engraved or loaded on a cylindrical roller, which is used to directly transfer ink through contact pressure to rolls of substrate, as shown in Figure 4c. In return for unmatched large‐scale production and cost‐effectiveness in the long run, alongside a lack of requirements on the flexibility of the substrate or properties of the ink, the cylindrical rollers and machinery costs render gravure printing costly to be employed in the initial phases, and far less accommodating to design changes in comparison to other printing fabrication methods.^42^ Combined with a R2R printing configuration, gravure printing is extremely suitable for high‐volume production, and considered ideal for industrial purposes.^49^

### 3.4 Aerosol Jet Printing

Aerosol jet printing is a relatively new printing technique developed in recent years for noncontact printing of flexible electronics. In this process, liquid inks are first atomized into small droplets of ≈1–5 µm in diameter, then transported to the printing head through a carrier gas. At the printing head, an annualer sheath flow is further used to aerodynamically focue the aerosol mist for jetting onto the target substrate (Figure 4d). This approach can push the printing precision down to the level of ≈10 µm, and can manufacture layers ranging from tens of nanometers to a few micrometers in thickness.^51^ Moreover, this technique is compatible with a wide range of materials, including high‐viscosity inorganic inks, organics, and high‐aspect‐ratio CNT solutions.^27^, ^52^ Another advantage of aerosol jet printing is its unique ability to print patterns on nonflat (3D) surfaces because of the large standoff distance between the printing nozzle and the substrate.^27^ In addition, the platen stage of the aerosol jet printer can be heated up to 200 °C for facilitating the evaporation of inks to achieve better printing quality, and avoid the coffee ring problem frequently suffered in inkjet printing. Recently, it has gained significant interest in the printing of transistors^52^, ^53^ as well as high frequency circuits and systems.^54^

### 3.5 Extrusion‐Based 3D Printing

The extrusion‐based 3D printing technique has been demonstrated in the multilayer fabrication of diodes.^41^, ^55^ In fused deposition modeling, the model layers are produced through the extrusion of small beads of material from a moving printhead, typically heated, which harden immediately to form the structure.^41^ Unlike other printing techniques which excel in scalability such as inkjet or gravure printing, 3D printing has low throughput and high operating costs.^23^, ^28^ In particular, the necessity of interweaving multiple materials into the fabrication process is still a major challenge, for which careful selection of the printing technique and material categories is required. Despite such issues, 3D printing represents one possible method of overcoming the nearly ubiquitous difficulty met by printing techniques in multilayer design fabrication. The research on the 3D printing of diodes has only recently begun to appear, and thus the drawbacks and advantages of this field, as well as the ideal fabrication methods and most suitable material combinations, remain relatively unexplored.

### 3.6 Postprocessing of Printed Materials

After the deposition of solution‐processed materials, especially in the case of inorganic materials, thermal annealing becomes an extremely important step to bringing out the maximum performance of the resulting device.^1^, ^53^ The function of the annealing is mainly twofold: one is to evaporate the solvent used in solution‐processing alongside other impurities to ensure ideal layer quality, and the other is to partially or fully melt the separated nanoparticles together to get better conductivity or chemically convert the deposited precursor to a stable semiconductor layer via thermal energy. Currently there are two major thermal annealing methods: i) conventional thermal annealing via furnace, oven, or hotplate;^52^, ^53^, ^56^ ii) photothermal annealing via laser or flash lamp.^57^, ^58^, ^59^ The first annealing approach has been widely used for most printing applications like transistors or sensors with printed electrodes from Ag/Au nanoparticle inks due to the low‐cost and simplicity in implementation of such a method.^52^ However, the drawback of this method is that in multiple layered devices, the curing of newly printed layers will lead to repeated curing of the former printed layers multiple times and even with different temperatures, which may generate negative effects on the affected layers as well as the device's performance, particularly when certain organic materials are involved.^60^ More significantly, some flexible substrates may not be able to survive at high curing temperatures, limiting the possible application of this general thermal annealing method.

Another annealing approach, termed photothermal annealing, relies on light to directly deliver thermal energy to the necessary layer.^57^, ^58^, ^59^ The most common method is to use a focused laser beam for the sintering. The highly localized energy from the laser can be controlled more accurately through adjustment of the environmental gases and the laser parameters, minimizing the negative impact to the target substrate and surrounding areas.^57^ Nevertheless, this method usually has a lower throughput due to the time‐consuming sequential process.^58^ Recently, UV light is also employed for the sintering of printed electronics. The short‐term UV light pulses emitted from xenon lamps can be tuned through the pulse duration, flash number, and power output parameters to maximize the thermal energy output to the target layer (>800 °C) while minimizing impact to the substrate.^61^ The xenon lamps can be combined with roll‐to‐roll based processes, resulting in large‐area processability and high efficiency.^59^ Due to the cost‐effective and environmentally friendly merits of photothermal annealing method, it has been attracting more attention and applications in the past few years.

## 4 Printing Materials and Their Applications for Diodes

### 4.1 Silicon

**Figure 5:**
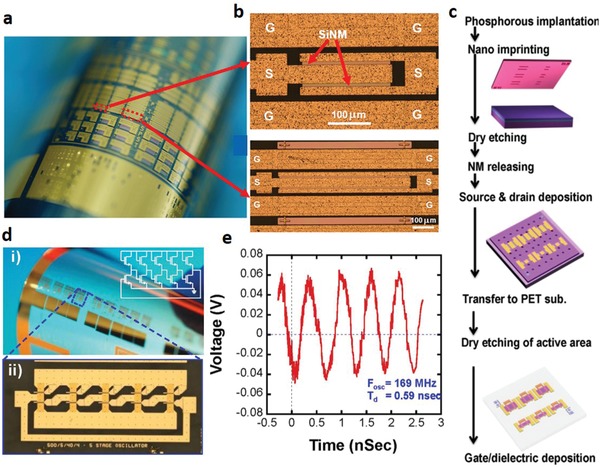
Printed diodes from silicon materials. a) Optical image of the finished PIN diodes arrays on a bent PET substrate. b) Microscopic image of the 80 mm^2^ flexible microwave single‐crystalline SiNM PIN diode on plastic substrate (top) and Microscopic image of the 240 mm^2^‐PIN diode on plastic substrate (bottom). Reproduced with permission.^64^ Copyright 2011, Elsevier. c) Schematic illustration of fabrication process for nanotrench Si NM flexible RF TFTs by NIL. d) i) A microscope image of a bent array of TFTs and ring oscillators on a PET substrate. ii) A microscopic image of a single 5‐stage ring oscillator under a flat condition. e) Measured voltage–time characteristic of the 5‐stage ring oscillator showing a frequency of 165 MHz and a delay time of 0.59 ns. Reproduced with permission.^65^ Copyright 2016, Springer Nature Group.

Single crystalline silicon is the material currently dominating the cutting edge of rigid RF electronics, possessing undoped electron and hole mobilities of 1400 and 450 cm^2^ V^−1^ s^−1^, respectively.^62^ However, it is difficult to utilize in flexible electronics due to the challenges of the preservation of its performance without severe degradation of scalability. In the fabrication of diodes, the transference of single crystalline Si nanomembranes (SiNMs) to flexible substrates has yielded superior results performance‐wise in comparison to solution‐processing methods.^1^, ^27^ For example, Qin and co‐workers have successfully employed transferrable SiNMs to fabricate flexible microwave P‐type‐Intrinsic‐N‐type (PIN) diodes capable of high‐frequency response at above 10 GHz,^63^, ^64^ as shown in **Figure** **5**a,b. More recently, Seo et al. further simplified the fabrication process by using nanoimprint lithography (NIL) to make flexible RF electronics with finely defined patterns, thereby potentially broadening RF applications (Figure 5c).^65^ The RF TFTs fabricated with NIL patterned deep sub‐microscale channel lengths and flexible Si NM on plastic substrates demonstrated a record‐breaking 38 GHz maximum oscillation frequency.^65^ They further showed a 5‐stage ring oscillator built on poly(ethylene terephthalate) (PET) with TFTs of 200 nm trench width and 20 µm channel width (Figure 5d), which exhibited an oscillation frequency of 169 MHz and corresponding stage delay of 0.59 ns, respectively (Figure 5e). However, the transference of SiNMs typically require highly complex steps such as ion implantation, photolithography, dry etching, and HF etching, as well as high temperature or high vacuum conditions. The lack of cost‐effectiveness and throughput has rendered SiNM‐based devices incapable of filling the void for low‐cost flexible RF electronics. Thus, great desire is invested in the development of new fabrication techniques offering higher scalability while compromising on performance, overlapping with the field of printed electronics. A general summary of the important performance parameters for the fabricated devices can be seen in **Table** **1**.

**Table 1:** Comparison of the performances of devices made of a variety of semiconducting materials reviewed in this paper. Although solution‐processed and printed diodes are given priority, other devices with procedures potentially applicable to printed diode fabrication or useful for comparison are alos included in the table. Note that in nonrectifying diode devices, the *V*_in_ parameter is used to indicate the turn‐on voltage

Device	Materials	Fabrication method	Frequency	*V*_in_ [V]	*V*_in_ [V]	Mobility [cm^2^ V^−1^ s^−1^]	Ref.
TFT	Si nanomembrane	NIL	5000	–	–	155–460	65
Schottky diode	Si microparticles	Screen, inkjet printing	1600	1	–	–	68
Schottky diode	Si microparticles	Lamination	1800	0.5	–	–	70
TFT, Poly‐Si	Si precursor cyclopentasilane	Spin‐coating, inkjet printing	–	–	–	6.5–108	71
TFT, a‐Si	Si precursor cyclohexasilane	Spin‐coating	–	–	–	<1.7	73
TFT, µc‐Si	Si precursor cyclohexasilane	Spin‐coating	–	–	–	–	75
Schottky diode	IGZO	Spin‐coating	–	<1.5	–	<10	88
TFT	IGZO	Gravure printing	–	–	–	0.81	86
TFT	IGZO	Spin‐coating	–	–	–	1.73	197
Seven‐stage ring oscillators–TFT	IGZO	Spin‐coating	340	–	–	7.0	81
TFT	IGZO	Spin‐coating	–	–	–	11.2	198
TFT	IGZO	“Sol–gel on chip”	–	–	–	7−12	60
TFT	IGZO	Spin‐coating	–	–	–	0.96	82
TFT	IGZO	Spin‐coating	–	–	–	2.0	83
Rectifier–Schottky nanodiode	ZnO	a‐Lith	20	±4	1.2	–	38
Schottky diode	IGZO	Sputtering	2450	–	–	–	199
Rectifier–Schottky diode	ZnO	Gravure printing	13.56	±5	4–4.5	–	94
TFT	ZnO	Spin‐coating	–	–	–	25–45	90
TFT	ZnO	Spin‐coating	–	–	–	4–11	89
TFT	ZnO	Spin‐coating	–	–	–	6	91
TFT	SWCNTs	Inkjet printing	18.21	–	–	6	133
Schottky diode	SWCNTs	DEP	18	0.4	–	–	134
TFT	SWCNTs	Aerosol jet printing	5	–	–	–	200
TFT	SWCNTs	Aerosol jet printing	5	–	–	–	201
Schottky diode	SWCNTs	Direct growth	540	–	–	–	107
Schottky diode	Graphene	DEP	26	–	–	–	147
Schottky Diode–Rectifier	Pentacene	Thermal evaporation	1240	±10	3.8	0.11	168
TFT transdiode	TIPS‐pentacene	Zone‐casting	1–9.8	–	–	0.03	169
TFT transdiode	C_10_‐DNBDT	Edge‐casting	1–22	±15	8	3.0	173
Schottky Diode–Rectifier	C_16_IDT − BT	Spin‐coating	>>13.56 (<68)	±20	5.1	1.5–2.5	177
MIS diode	PTAA	Inkjet printing	–	±10	–	–	178
Schottky Diode–Rectifier	PTAA	Gravure printing	>13.56	±10	3.5	–	11
Schottky Diode–Rectifier	PQT‐12	Spin‐coating	>13.56	±10	4	0.1–0.7	175
Schottky Diode–Rectifier	PTAA	Gravure printing	10	±10	2.7	–	174

#### 4.1.1 Silicon Particles

**Figure 6:**
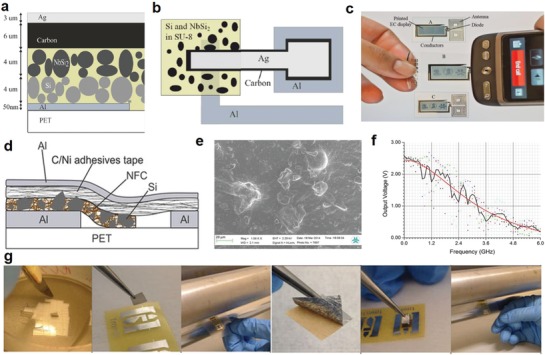
Flexible diodes from silicon particles. Schematic illustration of a) the cross section and b) the lateral architecture of the Schottky diode made of two layers of microparticles (NbSi_2_ and Si) bonded together by SU‐8 binder. c) Demonstration of e‐label application. The antenna–diode–display circuit is deposited onto a PET substrate. When the mobile phone is held close to the antenna, the display starts to switch on. Reproduced with permission.^68^ Copyright 2014, National Academia of Science. d) Illustration for the structure of the modified Schottky diode. e) SEM image of top NFC:Si film surface. f) Altered response of output DC voltage to signal frequency. g) The fabrication process of the diode: Peeling off the Si film; Attaching it to the substrate; Calendering; Peeling off the Ni/C double side adhesive tape; Attaching the Ni/C tape to the Si film; Calendering once more. Reproduced with permission.^70^ Copyright 2016, Springer Nature Group.

Silicon microparticles, defined as particles of Si between 0.1 and 100 µm in diameter, have been used for developing printed solar cells in the last decade with the dispersion of Si suspension inks.^66^, ^67^ However, the exploration of Si microparticles for printed diodes has recently attracted a great deal of interest.^1^, ^68^, ^69^ In particular, Sani et al. demonstrated a printed rectifier incorporating Schottky diodes capable of operation at 1.6 GHz (**Figure** **6**a–c).^68^ In their fabrication, a 4 inch n‐doped Si wafer with a doping level of 10^18^ cm^−3^ was processed through ball milling into microparticles 0.7 µm in diameter on average. As shown in Figure 6a, these particles were bonded in an organic polymer binder (SU8) to ensure charge carriers would only flow through a few individual particles, thus eliminating electron mobility losses due to electron charge scattering. The resulting 4–5 µm layer was stacked with an additional conducting buffer layer of similar‐size NbSi_2_ particles in the same polymer binder, an oxide‐free conduction layer of carbon to introduce current rectification, as well as two electrode layers on the top and bottom on a PET substrate to form the structure PET/Al (50 nm)/Si&SU8 (cross‐linked, <5 µm)/NbSi_2_&SU8 (<5 µm)/carbon (6 µm)/Ag (3 µm), as shown in Figure 6a. An additional Al‐foil antenna structure as well as an electrochromic display based on the poly(3,4‐ethylenedioxythiophene):polystyrene sulfonate (PEDOT:PSS) polymer were included on the PET substrate (Figure 6b), and the resulting e‐label demonstrated remote signal and power transfer in concert with a smart phone successfully (Figure 6c). This success directly elevated Si microparticles to the most promising material in the fabrication of Si flexible electronics, possessing a balance of adequate performance and suitability for commercial mass production.

More recently, the researchers from the same group presented a simplified fabrication procedure capable of producing Schottky diodes of up to 1.8 GHz (Figure 6d–g).^70^ In place of SU8, nano fibrillated cellulose (NFC) and glycerol were mixed with Si microparticles and water to form a nanocellulose–silicon composite film (Figure 6e), which was then laminated between a prepatterned bottom Al electrode layer and a conductive carbon tape top contact coated in Ni to form the structure PET/Al/NFC&Si (10–15 µm)/Ni:carbon (Figure 6d). The semiconducting film is highly reconfigurable, being easily delaminated from the PET/Al substrate for transference to other substrates, and the entire fabrication procedure, as shown in Figure 6g, possesses excellent potential for mass production due to a lack of temperature constraints while only requiring a calender machine for pressing and laminating the different film layers together.

#### 4.1.2 Solution‐Processed Silicon

Silicon is extremely difficult to utilize in solution processing due to the challenge of maintaining the superb electrical properties of single crystalline silicon in a solution form. The prevalent use of silane‐based liquid as a precursor for conversion to high purity amorphous silicon (a‐Si) or microcrystalline/nanocrystalline silicon (µc‐Si/nc‐Si) has limited compatible precursors to hydrogenated silicon compounds of the straight‐chain (Si*_n_*H_2_ *_n_*_+2_) or cyclic forms (Si*_n_*H_2_ *_n_*).^71^ Moreover, the pyrolysis procedure in the process requires high temperature, resulting in incompatibility with most flexible substrates, and the production of harmful monosilane gases. Even then, solution‐processed silicon has limited carrier mobilities and faces great difficulty in achieving operation at the UHF band.^71^ However, unlike organic materials, solution‐processed silicon can experience enhancement in semiconductor properties before and after layer formation by doping, while silicon possesses longer lifetime and negligible degradation, which may render it an available option for printed RF diodes.^67^, ^72^

The procedures for converting the hydrosilane precursor compound to a printed silicon layer involve four steps: polymerization, printing, pyrolysis, and optional annealing. Silicon ink is composed of a chain‐like polydihydrosilane and an organic solvent such as toluene.^73^ To convert the hydrosilane precursor to polydihydrosilane, UV light irradiation or sonification can be applied to the precursor to trigger the ring‐opening polymerization.^74^ The resulting hydrogenated polysilanes are diluted once more with the monomer precursor and an organic solvent, then filtered to remove the insoluble polysilanes that precipitated in the dilution process to obtain “liquid Si.”^71^ Following ring‐opening polymerization and the formation of ink, a printing process such as inkjet or screen printing can be used to deposit a layer of the ink, which would stabilize into a polydihydrosilane film coated in a thin SiO_2_ layer due to oxidation.^72^ Finally, the polydihydrosilane layer can be transformed into pure amorphous silicon through the process of pyrolysis, in which heat is applied to induce thermal decomposition. If conversion from a‐Si to µc‐Si is desired, additional thermal and laser annealing steps may be employed, while ambient hydrogen plasma treatment can further enhance the Si layer's electronic properties.^71^, ^75^

**Figure 7:**
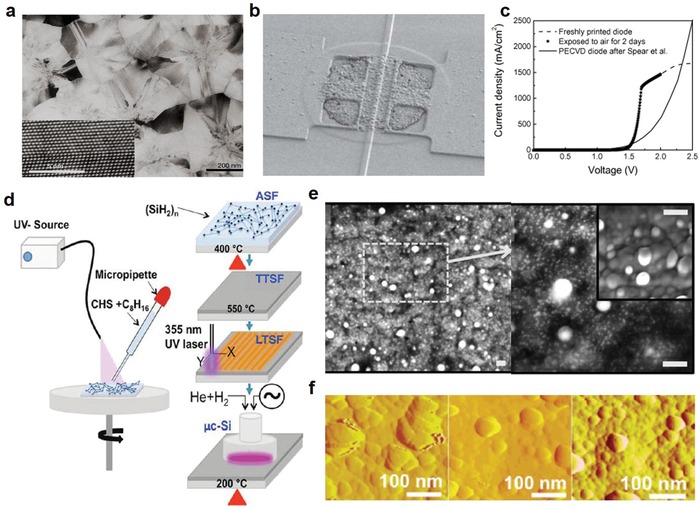
Printed electronics from solution‐processed silicon. a) A TEM image of a solution‐processed poly‐Si film. The film was formed by spin‐coating and baking of the liquid silicon materials followed by laser crystallization. The inset TEM image highlights the atomic image of the silicon crystal. The grain size in the film is about 300 nm, which is comparable to that of conventional CVD‐formed poly‐Si film. b) SEM image of a TFT made from ink‐jet printed silicon film. Reproduced with permission.^71^ Copyright 2006, Springer Nature Group. c) Comparison of current density–voltage (JV) characteristic of printed and plasma‐enhanced chemical vapor deposition (PECVD) based a‐Si diode. Dashed and star curves denote freshly printed and air aged printed diodes, respectively. Reproduced with permission.^73^ Copyright 2008, Elsevier. d) Schematic of the solution‐based synthesis of microcrystalline silicon (µc‐Si) thin films. e) SEM images of a cyclohexasilane precursor‐based Si film after plasma hydrogenation treatment for 20 min. Each scale bar is 100 nm. f) Changes in grain size and roughness of the surface in (e) following plasma hydrogenation treatment of 0, 20, and 30 min (left to right). Reproduced with permission.^75^ Copyright 2015, American Chemical Society.

Among the potential hydrosilane compounds, cyclopentasilane and cyclohexasilane have been identified as suitable precursors. Both possess superior stability in comparison to other hydrosilane compounds, maintaining relatively lower vapor pressure while remaining stable under ambient light, and cyclopentasilane in particular is noted to possess a high photoreactivity on UV light irradiation.^71^, ^75^ Initial work with cyclopentasilane was first presented by Shimoda et al.,^71^ in which the solution‐processed polycrystalline silicon was combined with printing for the fabrication of flexible electronics (**Figure** **7**a,b). Despite having yet to reach the performance of single crystalline silicon on rigid substrates, the mobilities exceeded most of those found in solution‐processed organic TFTs, as well as those of amorphous silicon TFTs.^71^ Meanwhile, Han et al. used a similar fabrication process with cyclohexasilane to form a Si ink which was converted to a‐Si after layer formation through spin coating.^73^ The as‐fabricated a‐Si PN junction diodes demonstrated excellent current density at low voltages in comparison to a‐Si PN junction diodes prepared by chemical vapor deposition (CVD), as shown in Figure 7c. They found that a‐Si could be generated through collimated aerosol beam deposition in lines as thin as 10 µm.^73^ Similarly, Iyer et al. further refined the use of thermal annealing, laser annealing, and especially plasma hydrogenation treatment following pyrolysis on solution‐processed Si for conversion from a‐Si to µc‐Si (Figure 7d–f).^75^ Their results demonstrated a potential conductivity in spin‐coated µc‐Si films of above 10^−1^ S cm^−1^ depending on the duration of H‐plasma treatment in the plasma hydrogenation step.^75^

### 4.2 Metal Oxide Semiconductors

MOS are typically n‐type, and provide relatively high performance as well as a superior oxygen stability.^1^, ^27^, ^76^ The optoelectronic properties of MOS have been widely used in LEDs, while their transparency has made them extremely attractive for display‐type applications.^28^ The chemical properties of MOS materials have rendered them far more suitable for solution‐processing than silicon, although the issues regarding the high annealing temperatures do still exist. However, the presence of grain boundaries in MOS materials and their low uniformity in printing have limited their applications in large‐scale fabrication.^1^ The selected fabrication process has a tremendous impact on the final performance and quality of metal oxide layers. Currently, the most common manufacturing techniques include physical vapor deposition, pulsed laser deposition, and atomic layer deposition.^12^, ^77^, ^78^ The representative solution‐processed, nonphotonic MOS devices fabricated with approaches that can be potentially applicable for printing diodes are summaried in Table 1.^79^

#### 4.2.1 Indium Gallium Zinc Oxide (IGZO)

Indium nitrate hydrate (In(NO_3_)_3_∙χH_2_O), gallium nitrate hydrate (Ga(NO_3_)_3_∙χH_2_O), and zinc acetate dehydrate (C_4_H_6_O_4_Zn∙2H_2_O) are the most prominent precursors selected for synthesizing IGZO with specific ratios due to their higher charge carrier concentrations in associated films.^80^, ^81^, ^82^, ^83^ It is discovered that IGZO layers can generally retain amorphous phase structure (without grain boundaries), high degrees of transparency, and maximum semiconductor performance in annealing temperatures of ≈300–400 °C.^78^, ^80^ Therefore, only substrate materials capable of withstanding high temperatures like silicon or glass may be used, excluding most flexible substrates such as PET and other polymers.^84^ For example, Kim et al. demonstrated inkjet printing and gravure printing of IGZO to fabricate TFTs possessing carrier mobilities of 0.03 and 0.81 cm^2^ V^−1^ s^−1^, respectively.^85^, ^86^ In the annealing process, the IGZO film and substrate were treated under high temperature to induce the chemical decomposition and dispersion of unnecessary materials, getting IGZO of high purity. With the same materials, Lim et al. optimized the precursor ratio, and revealed that in an In:Ga:Zn ratio of 1:*X*:1, the ideal gallium proportion was 0.5, through which mobility as high as 2 cm^2^ V^−1^ s^−1^ could be obtained at an annealing temperature of 400 °C.^83^

**Figure 8:**
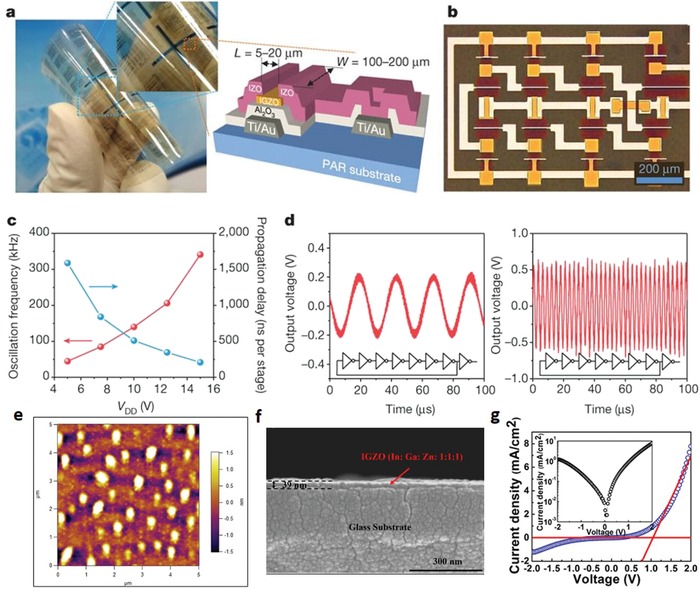
Flexible electronics from IGZO. a) Optical image and a schematic cross‐section of photoannealed IGZO TFTs and circuits on PAR. b) Optical image of a seven‐stage ring oscillator. Gate to source/drain overlap distance is 5 µm. c) Oscillation frequency (red) and per‐stage propagation delay (blue) of the ring oscillator as a function of supply voltage, *V*_DD_. d) Output waveforms of the ring oscillator operating with supply voltages of 5 V (left panel) and 15 V (right panel), and oscillation frequencies of 45 and 341 kHz, respectively. Reproduced with permission.^81^ Copyright 2012, Springer Nature Group. e) AFM image and f) cross section SEM image of IGZO (In:Ga:Zn = 1:1:1) composition film, annealed at 400 °C for 2 h. g) *I*–*V* curves of IGZO based heterojunction didoes. Reproduced with permission.^88^ Copyright 2017, Elsevier.

Remarkably, Kim et al. proposed a room‐temperature process to achieve condensation and densification of a‐IGZO layers through photochemical activation.^81^ The samples were heated up to 150 °C by the transmitted energy of the deep‐ultraviolet rays, which could assist in the removal of organic residues from the precursor. Using this method, they successfully fabricated a‐IGZO TFTs with carrier mobility up to 7 cm^2^ V^−1^ s^−1^ on a flexible polyarylate (PAR) substrate (**Figure** **8**a).^81^ To demonstrate device scalability, the authors also fabricated seven‐stage ring oscillator circuits using room‐temperature‐fabricated IGZO TFTs on the PAR substrates (Figure 8b). The results showed that with a supply voltage of *V*_DD_ = 15 V, an oscillation frequency larger than ≈340 kHz, and corresponding propagation delay less than ≈210 ns per stage (Figure 8c,d) could be obtained.

Recently, Su et al. presented a similar photoannealing method to obtain a field‐effect mobility of 1.73 cm^2^ V^−1^ s^−1^ for TFTs by means of ultraviolet‐ozone treatment of the sample at 300 °C, superior to thermally annealed devices with the same precursor solution (In:Ga:Zn = 0.63:0.05:0.32).^87^ Banger et al. also developed a “sol–gel on chip” hydrolysis approach from soluble metal alkoxide precursors to form amorphous metal oxide semiconducting thin‐films.^60^ They utilized aqueous hydrolysis annealing on a hot plate at maximum process temperatures as low as 230 °C to fabricate highly stable IGO and IGZO TFTs with field‐effect mobilities of 7–12 cm^2^ V^−1^ s^−1^, which were comparable with those similar TFTs made through sputtering.^60^ The process is applicable to a broad range of amorphous MOSs, and should provide new opportunities for integrating amorphous oxide materials into functional electronic and optoelectronic devices.

At present, most flexible MOS‐based diodes operating in the HF and UHF range are based on IGZO materials, and typically fabricated through sputtering deposition. Rare solution‐processed diodes made from IGZO were reported. One recent example was the fabrication of p–n junction diodes via solution‐processing of IGZO as the n‐type semiconductor and CuO as the p‐type semiconductor.^88^ A mixing ratio of 1:1:1 was selected for In:Ga:Zn, and after spin‐coating and curing of IGZO (Figure 8e,f), an additional CuO layer was added on top of the IGZO layer. For compatibility with the flexible polyimide substrate, UV exposure was utilized at room temperature in a nitrogen environment after preannealing the IGZO films at a lower temperature of 325 °C. In such a process, only a slight increase of roughness was observed without much impact upon other properties (Figure 8e,f), suggesting UV treatment of the films may be an effective means to lower the processing temperature. Figure 8g depicts the characteristics of a representative diode, indicating the turn on voltage (*V*_ON_) of 1.11 V and the rectification ratio (*I*_on/off_) of 20.^88^

#### 4.2.2 Other Metal Oxides

**Figure 9:**
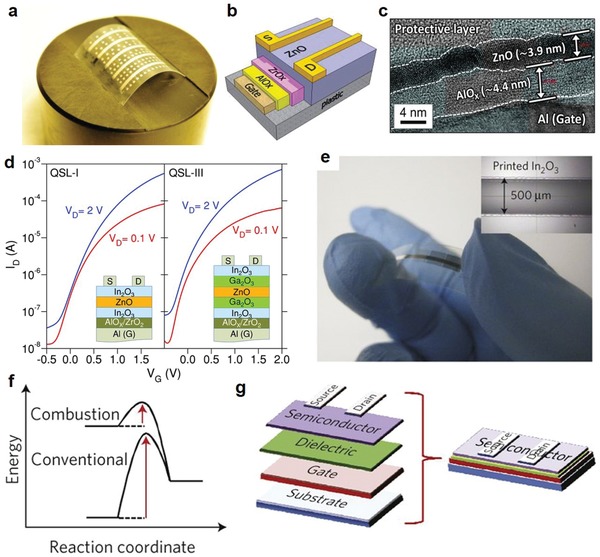
Flexible transistors and diodes based on solution‐processed metal oxides. a) Photograph of an actual transistor array containing 35 flexible ZnO TFTs based on a UV‐grown bilayer AlO*x*/ZrO*x* gate dielectric and Al source–drain electrodes. b) Schematic of the device architecture used and c) TEM image of the SiO2/ZnO/Al cross‐section showing the ultrathin nature of the ZnO film. Reproduced with permission.^89^ Copyright 2013, Wiley‐VCH. d) Representative sets of transfer characteristics measured from transistors based on metal oxide quasi‐superlattices (QSL) channels. Reproduced with permission.^90^ Copyright 2015, Wiley‐VCH. e) Optical image of a flexible combustion‐processed In_2_O_3_ device on AryLite (30 nm Al gate electrode/41 nm a‐alumina dielectric, with 30 nm Al source and drain electrodes) and optical image of an inkjet printed In_2_O_3_ line on n++Si/41 nm a‐alumina (inset). f) Comparison of energetics of combustion synthesis‐based processes versus conventional approaches. g) schematic of top‐contact bottom‐gate TFT device structure in (e). Reproduced with permission.^91^ Copyright 2011, Springer Nature Group.

The solution‐processing of other metal oxides such as ZnO, IGO, and In_2_O_3_ is similar to that of IGZO. As shown in **Figure** **9**a,b, Lin et al. successfully fabricated ZnO transistors on temperature‐sensitive polyethylene naphthalate (PEN) substrates with field‐effect mobilities of 4–5 cm^2^ V^−1^ s^−1^ after annealing in temperatures up to 160 °C.^89^ This was achieved by using a carbon‐free molecular ZnO hydrate precursor to form high‐quality polycrystalline ZnO layers ≈4–5 nm in thickness (Figure 9c). Further work was performed to exploit the better electron transport properties of low‐dimensional polycrystalline heterojunctions and quasi‐superlattices (QSLs) through depositing alternating layers of In_2_O_3_, Ga_2_O_3_, and ZnO of less than 10 nm thick on glass substrates (Figure 9d) by sequential spin casting of different precursors in air at low temperatures (180–200 °C).^90^ Optimized prototype QSL transistors exhibited band‐like transport with electron mobilities approximately ten times greater (25–45 cm^2^ V^−1^ s^−1^) than single oxide devices (typically 2–5 cm^2^ V^−1^ s^−1^), indicating a promising perspective of this method for application in next‐generation large‐area optoelectronics and large‐area microelectronics such as ultrahigh definition optical displays.

Significantly, Kim et al. introduced a completely divergent tactic, using a redox‐based combustion chemical technique generated through the precursor solution itself to perform the annealing step (Figure 9e–g).^91^ By using acetylacetone or urea as fuel and metal nitrates as oxidizers, a localized, self‐energy‐generating exothermic reaction can trigger conversion of the precursor solution to the MOS layer without excessive spread of heat to the substrate (Figure 9f). This strategy was successfully demonstrated with In_2_O_3_, a‐Zn‐Sn‐O, a‐In‐Zn‐O, and indium tin oxide (ITO) to yield a maximum electron mobility of ≈6 cm^2^ V^−1^ s^−1^ with processing temperature of around 200–250 °C.^91^

**Figure 10:**
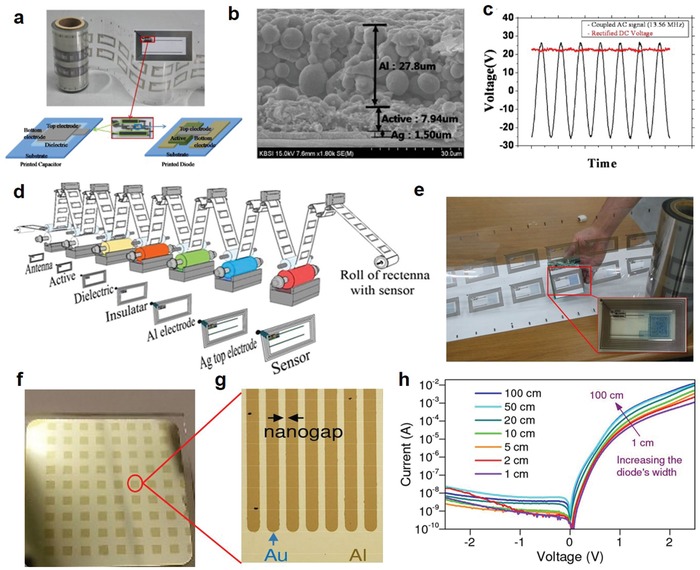
Printed diodes with metal oxides. a) Optical image of the R2R gravure printed rectenna on PET foil. The bottom schematics describe the components of rectenna: printed capacitor, printed diode, printed bottom Ag electrode. b) Cross‐sectional SEM images for the R2R gravure printed diode. c) Input–output electrical characteristics of rectifier at 13.56 MHz A, indicating a rectifying efficiency of about 90%. Reproduced with permission.^93^ Copyright 2012, IOP Publishing. d) Scheme for R2R gravure to completely print sensor‐Signage Tags on PET foils. e) R2R samples by combining R2R gravure printed rectenna and R2R coated electrochromic signage. Reproduced with permission.^94^ Copyright 2014, Springer Nature Group. f) Photograph of a 2 × 2 cm^2^ substrate patterned with the use of the adhesion lithography (a‐Lith) technique. The substrate incorporates 72 discrete Al/Au nanogap diodes. g) Optical micrograph of a‐Lithfabricated interdigitated electrode structures. h) Current–voltage characteristics of Al/ZnO/Au nanogap diodes (with different widths) fabricated on the substrate shown in (f). Reproduced with permission.^192^ Copyright 2016, SPIE.

The printed MOS diodes that can operate at the 13.56 MHz band have been successfully used in RFID‐based applications.^92^ For example, Park et al. used a R2R gravure printing process to fabricate a 13.56 MHz rectenna with layer‐by‐layer printing of ZnO semiconducting ink as the active layer for the diode, Ag conductive ink for the antenna and one electrode, Al‐based conductive ink for the top electrode of the diode, BaTiO3‐based ink for the dielectric layer of the capacitor, and epoxy‐based ink for the insulating layer on a PET foil substrate (**Figure** **10**a,b).^93^ The all R2R gravure printing process was performed under a roll pressure of 0.8 MPa and a speed of 8 m min^−1^ at annealing temperatures no more than 150 °C.^93^ The high rectifying efficiency of one printed diode and capacitor can reach about 90% at 13.56 MHz, as shown in Figure 10c, indicating almost total conversion of AC signal into DC voltage. Further work by this group successfully incorporated this rectenna circuit into a wireless sensor‐signage tag compatible with R2R gravure printing (Figure 10d) alongside a humidity sensor and an electrochromic signage unit on a PET film (Figure 10e), opening wide the gateway to the use of printed diodes in inexpensive HF wireless power transmission.^94^

Most recently, Semple et al. successfully fabricated ZnO‐based coplanar Schottky nanodiodes on glass and PET substrates.^36^, ^37^ Utilizing adhesion lithography (a‐Lith), they achieved electrode spacing less than 20 nm in an unprecedented lateral architecture (Figure 10f,g).^38^ This architecture brings a variety of benefits aside from its low surface area, including low device series resistance, negation of scattering at ZnO grain boundaries, and stable device performance. As shown in Figure 10h, despite a large electrode width, the fabricated coplanar nanogap diodes could operate at low voltages (±2.5 V) and were able to sustain a high forward current of 10 mA while the reverse current remained low, on the order of 10 nA.^38^

### 4.3 Nanomaterials

Nanomaterials, such as CNTs, graphene, 2D materials, and quantum dots, have been receiving increased intention in semiconductor devices including diodes over the years. The unique structures of these carbon‐based nanomaterials allow themselves to outperform all competitors in operation frequency and current densities with near‐certainty, although some severe challenges in fabrication, integration, and scalability remain in developing various kinds of devices and applications. A summary of the parameters and performance for nonphotonic devices is presented in Table 1. While quantum dots have found great success in photonic LED devices, the carbon‐based nanomaterials are characterized by exceedingly high semiconductor properties which no other material class may match.

#### 4.3.1 Carbon Nanotubes

Since their discovery in 1991, CNTs have been intensively studied and explored for nearly every type of electronics due to their superior mechanical, thermal, and electrical properties.^95^, ^96^ CNTs incorporated within diodes have been associated with relatively high breakdown voltages due to minimal self‐heating,^97^ while very little quantum capacitance has been observed due to their low dimensionality.^1^, ^98^ These superlative parameters have inevitably elevated CNTs to one of the most popular and competitive semiconductor materials in future high‐speed electronic devices.

**Figure 11:**
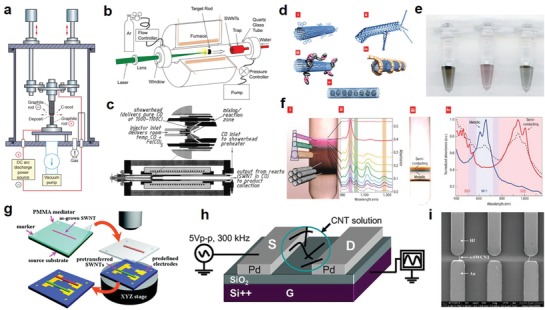
Schematic illustration of the major CNT synthesis techniques compatible with solution processing: a) arc‐discharge, b) laser ablation, and c) high‐pressure carbon monoxide growth. Reproduced with permission.^105^ Copyright 2004, Elsevier. The CNTs purification approaches for removing the mixed metallic CNTs: d) functionalization via polymer wrapping, e) chromatography and f) density‐gradient ultracentrifugation. Reproduced with permission.^109^ Copyright 2008, Springer Nature. The alignment methods for CNT applications in high performance devices: g) selective positioning via SEM/Raman spectroscopy. Reproduced with permission.^112^ Copyright 2009, American Chemical Society. h,i) Alternating current dielectrophoresis (DEP). Reproduced with permission.^115^ Copyright 2014, IEEE.

One grand challenge to hinder the wide applications of CNTs is the lack of an economically viable and high‐throughput production technique for CNTs.^99^, ^100^ Current fabrication techniques include arc‐discharge (**Figure** **11**a),^101^ chemical vapor deposition,^100^, ^102^ laser ablation (Figure 11b),^103^ and gas‐phase catalytic growth by carbon monoxide (Figure 11c),^104^ all of which are not ready for large‐scale mass production due to the prohibitive costs and substandard efficiency.^96^, ^105^ Another big obstacle for using CNTs in diodes and other semiconductor devices is the presence of metallic tubes in every fabricated batch.^106^, ^107^, ^108^ In the past decades, a few promising methods have been developed to purify the semiconducting single‐walled CNT (SWCNT) for inks used in printing electronics, including functionalization via polymer wrapping (Figure 11d), chromatography (Figure 11e), and density‐gradient ultracentrifugation (Figure 11f).^106^, ^109^, ^110^ However, significant effort is required to enhance the purification throughput and reduce the cost to the levels reasonable for mass production.

Furthermore, the electrical performance of CNTs is greatly affected by their alignment due to their typical tubular structure. Thus, controlling the alignment of CNT assembly and patterning becomes another difficulty for developing high‐performance CNT‐based electronics. “Blind” deposition without alignment control was used in a photolithographic route and confirmed to result in a low yield of functional devices.^111^ By contrast, as shown in Figure 11g, Jiao et al. utilized scanning electron microscopy (SEM) and Raman spectroscopy to selectively position individual SWCNTs with ultrahigh precision for perfect alignment, but this method completely discarded the hope of scalability or commercial viability.^112^ With alternating current dielectrophoresis (AC‐DEP), as shown in Figure 11h,^113^ in conjunction with photolithographic patterning, Li et al. reported HF Schottky diodes possessing excellent alignment (Figure 11i).^114^, ^115^ Similarly, Stokes et al. demonstrated the successful alignment of ultrahigh‐density SWCNT arrays using the DEP method, reaching a maximum density of 30 SWCNT per µm for aligned arrays.^116^

The dispersion of CNTs within a solvent to form desirable ink is a critical step in any solution‐based fabrication process. The ink parameters such as stability, material quality, dispersion degree, surface tension, and viscosity must be carefully optimized with regard to the selected printing method, substrate material, drying procedure, and platen temperature for achieving maximum device performance.^47^, ^117^ However, CNTs possess high interstructure interaction energy due to internal van der Waals forces, thus rendering dispersion in liquid difficult.^118^ In addition, the high aspect ratio of CNTs makes them prone to clumping and entanglement.^119^ Therefore, different methods have been explored for dispersing CNTs into a stable and uniform solution ink.

**Figure 12:**
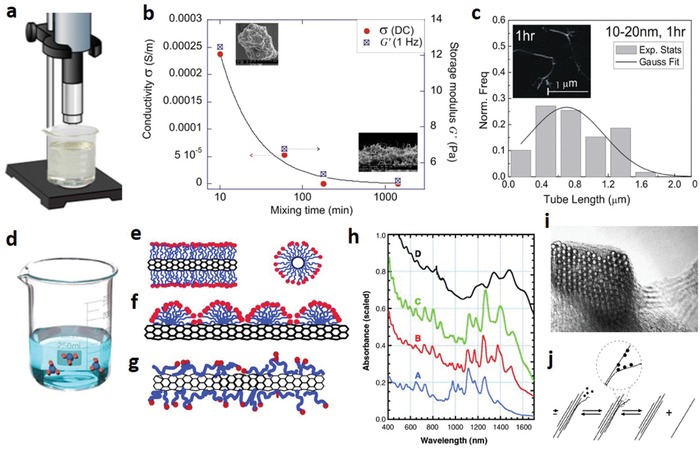
Methods for dispersing CNTs into a stable and uniform ink. a) Schematic diagram of the sonication procedure for CNTs. b) Plot of the decrease in CNT conductivity with increased physical mixing time. c) CNT length distribution after 1 h of sonication treatment (inset: SEM image of lightly damaged CNTs). Reproduced with permission.^193^ Copyright 2012, MDPI. d) Schematic of the functionalization procedure for CNTs. e) Cylindrical miscelle configuration of surfactants encapsulating a SWCNT. f) Hemimicellar adsorption configuration of surfactants on a SWCNT. g) Random adsorption configuration of surfactants on a SWCNT. h) Adsorption spectra of SWCNTs in an SDS surfactant suspension. Lines A–C represent SWCNTs of different diameters, while line D represents aggregated SWCNTs bundles. i) TEM cross‐sectional image of a SWCNT bundle. j) Separation procedure through which surfactants may disperse SWCNT bundles. Reproduced with permission.^125^ Copyright 2006, Elsevier.

Ultrasonication (**Figure** **12**a) has been intensively used to disperse CNT bundles within a solvent and evenly spread them throughout.^120^ Although ultrasonication is cost‐effective, easy to use, and reliable, it can cause damage to internal CNT structures ranging from defects or deformation in the cylindrical formation to the breakage, fragmentation, and shortening of CNTs, as shown in Figure 12b,c, negatively impacting the CNTs' performance.^121^ In addition, sonication may also negatively affect the solvents and dispersants in the solution, resulting in alteration of physical and chemical properties together with potential ink instability depending on the incorporated materials.^122^

The chemical functionalization of CNTs or the alteration of their surface properties for higher solubility through chemical moieties is also employed for the dispersion of CNTs.^123^ Despite being far less damaging to the nanotube structures than physical dispersion methods, high‐temperature functionalization utilizing aggressive chemicals like acids may cause defects in CNT structures or negatively impact their semiconductor properties.^124^ Adding surfactants is perhaps the most commonly utilized method in solution‐dispersed CNT inks to increase solubility and reduce clumping (Figure 12d). Figure 12e–g shows the main mechanism of surfactants in dispersing SWCNTs in different solvents.^125^ The dispersion process can be monitored by checking transient fluorescent emission as a function of various parameters, like the type of surfactant used, sonication time, as well as surfactant concentration and functionalization (Figure 12h–j).^125^ A great variety of polymers and surfactants have been tested for adsorption and bonding on the nanotube surface. Depending on the nature of the surfactant, its concentration, the identity of the solvent, the role of the stabilizing polymers, the treatment of the surface of the substrate, and other parameters such as solution temperature, the properties of the resulting CNT ink may vary greatly, requiring highly precise tuning to optimize ink parameters for each device application and printing method.^126^

**Figure 13:**
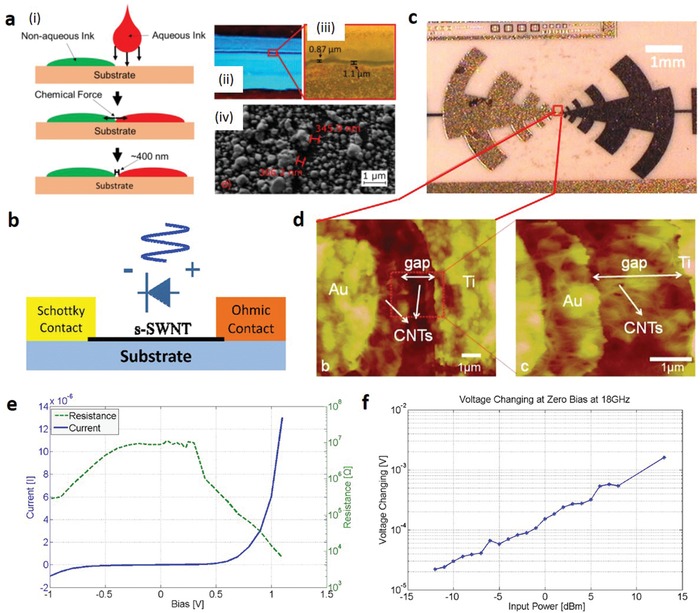
CNT‐based Schottky diode. a) Illustration of a fabrication approach using the chemical forces between inks. i) Schematic description for inkjet printing transistors with small channel gaps via chemical forces. ii,iii) optical images of two parallel lines forming a chemical gap. The lines are electrically isolated. iv) SEM image of an electronically isolated gap. Reproduced with permission.^133^ Copyright 2017, Springer Nature. b) Illustration for a CNT based Schottky diode detector. c) Photomicrograph of a log‐periodic antenna with CNT Schottky diodes, d) AFM scan of the CNT channel region of the diode, and e) close‐up view of CNT rich region. e) *I*–*V* characteristics for the Schottky diode of (b) alongside its effective resistance. f) Output voltage of the diode at 18 GHz as a function of input power. Reproduced with permission.^134^ Copyright 2014, IEEE.

The deposition of CNT thin films for flexible electronics has been performed by a number of different printing methods, including inkjet printing,^127^ aerosol jet printing,^52^, ^53^, ^56^, ^128^ gravure printing,^129^ screen printing,^130^ and transfer printing.^131^ Though CNTs have rarely been used in printed CNT diodes up to now, the performance of other devices has absolutely shown the great promise of this material in this field.^132^ For example, Grubb et al. printed CNT‐based TFTs on a Kapton substrate by inkjet printing, with very small channels (≈1 µm) formed by employing the chemical forces between inks (**Figure** **13**a).^133^ High‐purity SWCNTs were added to a proprietary nonaqueous solution possessing similar properties to N‐cyclohexyl‐pyrrolidone (CHP) at a ratio of 20% concentration by weight, and then dispersed through sonication. After inkjet printing, a thermal annealing procedure was applied to remove the proprietary solution. The printed transistors finally were capable of reaching operation speeds up to 18.21 GHz.^133^

While having yet to see significant results in printing, many CNT‐based diodes fabricated by other methods do exist, operating in the UHF, microwave, and terahertz bands. For instance, Yang and Chahal developed CNT Schottky diodes with the unique undercut and self‐alignment method.^134^ In their work, the DEP technique was used to align SWCNTs in between asymmetric, closely spaced (1 µm) electrodes to form the coplanar device structure (Figure 13b). A polyether ether ketone (PEEK) substrate was used due to its high compatibility with chemicals used in processing and its low loss‐characteristics at high frequency. Alignment of multiple CNTs in parallel between the electrodes allows for better impedance control for RF circuit designs. As an example, multiple CNTs are incorporated within a log‐periodic structure (Figure 13c,d). Using a coplanar structure, a rectified voltage of 9 mV is demonstrated at the strongest nonlinear region of the *I*–*V* curve (0.4 V) with input RF power of 13 dBm at 18 GHz (Figure 13e,f).^134^ Also, the equivalent model derived from this structure shows that the diode has high cut‐off frequency well into the terahertz (THz) region,^135^ highlighting the potential of CNT diodes for RF and THz circuit applications.

#### 4.3.2 Graphene

**Figure 14:**
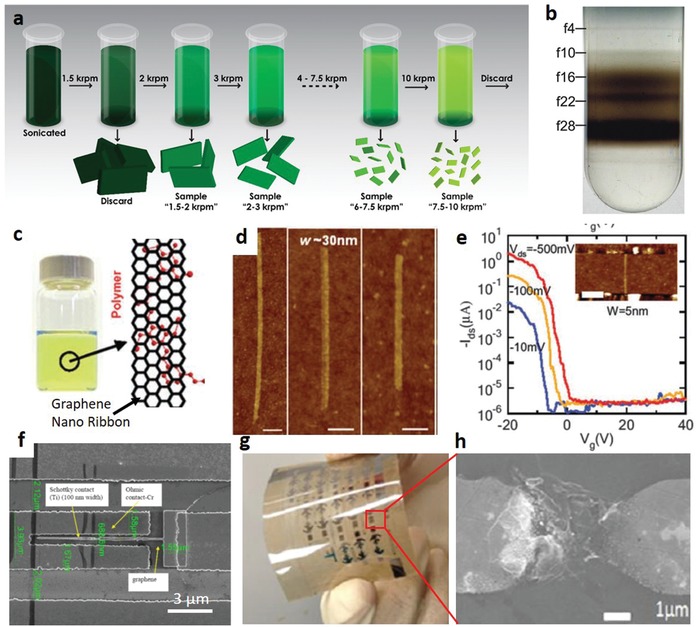
Current techniques in fabricating graphene nanosheets and Schottky diodes fabricated with graphene. a) Cascade centrifugation. Reproduced with permission.^194^ Copyright 2016, American Chemical Society. b) Density gradient ultracentrifugation Reproduced with permission.^195^ Copyright 2009, American Chemical Society. c) Photograph of a polymer PmPV/DCE solution with graphene nanoribbons stably suspended in the solution. d) Chemically derived graphene nanoribbons down to sub 30 nm width. e) Transfer characteristics of the transistor made of graphene nanoribbon with Pd contacts. (Inset) The AFM image of this device. Scale bar is 100 nm. Reproduced with permission.^143^ Copyright 2008, AAAS. f) SEM image of a Schottky diode fabricated on a graphene monolayer. Reproduced with permission.^146^ Copyright 2012, APS. g) Partially reduced graphene oxide‐based Schottky diodes on a PEEK substrate. h) Magnified SEM image of a single Schottky diode from (d). Reproduced with permission.^147^ Copyright 2013, IEEE.

Graphene, a monolayer 2D honeycomb lattice formed from carbon atoms, possesses optoelectronic, plasmonic, mechanical, and electrical properties highly valued in many applications.^136^ Like CNTs, graphene is a material deemed to possess incredible potential for electronics. Existing synthesis methods for graphene mainly include mechanical, solution‐based or chemical‐assisted exfoliation,^137^, ^138^ chemical synthesis,^139^ epitaxial growth,^140^ and pyrolysis.^138^, ^139^ From the perspective of solution‐processing and ink production, the most suitable synthesis route with potential for cost‐effective mass production lies in solution‐based exfoliation techniques on flakes of 2D graphene (**Figure** **14**a,b).^141^

The lack of a bandgap in graphene makes it difficult to utilize in semiconducting applications, including diodes. Thus far, the only possible solutions are to use partially reduced graphene oxide (rGO)^142^ or to open a bandgap through forming graphene nanoribbons.^143^, ^144^ However, chemically rGO must undergo a variety of thermal and chemical treatments to retain a diminished degree of electrical conductivity, but is noted to possess a highly disordered structure and electrical properties which are extremely difficult to tune. On the other hand, the carrier mobility and bandgap observed in graphene nanoribbons depend on the width of the ribbon, and can be comparable to those of CNTs.^145^ For example, Dai and co‐workers developed a chemical route to produce graphene nanoribbons with width below 10 nm, stably suspended in solvents with noncovalent polymer functionalization (Figure 14c,d). After the purification process, they found that all the sub 10 nm graphene nanoribbons produced were semiconductors and afforded graphene FETs on–off ratios of about 10^7^ at room temperature (Figure 14e).

Due to the lack of a bandgap, there are few graphene Schottky diodes reported up to date, and most operated only at low frequencies and exhibited poor performances.^1^ In a recent work, Dragoman et al. reported a Schottky‐like diode able to withstand currents at mA level and operate at millimeter wave frequencies, greatly surpassing corresponding currents in CNT‐based Schottky diodes.^146^ To achieve higher performance, they used a graphene monolayer with asymmetric metallic contacts deposited on a high‐resistivity Si substrate (Figure 14f). Remarkably, Chahal and co‐workers demonstrated solution‐processed flexible Schottky diodes having high cut‐off frequency using reduced graphene oxide (Figure 14g).^147^ By adding hydrazine hydrate to a graphene oxide dispersion to reduce the required annealing temperatures to 100 °C, the use of a thin polymer substrate (PEEK) was enabled on which the ink was deposited and aligned between Ti (Schottky) and Pd (ohmic) contact electrodes via the DEP technique (Figure 14h). As the graphene was not fully reduced, a bandgap was left to ensure the formation of the Schottky barrier, sacrificing carrier mobility in the process. Even so, the final device demonstrated frequency operation up to 26 GHz and could output a 2 mV DC voltage given a 10 dBm input power.^147^

#### 4.3.3 Quantum Dots

QDs, defined as very small semiconductor particles only a few nanometers in diameter, are believed to be a good candidate for the light‐emission layers of LEDs. The small size of QDs leads to their optical and electronic properties which differ from those observed in bulk materials.^148^ As individual QDs with varied sizes can naturally produce monochromatic light of specific gaussian distributions, greater precision and efficiency can be achieved due to the lack of necessity for color filtration with a larger spectrum of colors. A weakness does exist in the production of blue light, as most high‐quality blue QDs possess peak emission wavelengths below 455 nm.^149^, ^150^ This would result in weaker luminescence for shades of blue in comparison to other display technologies.

**Figure 15:**
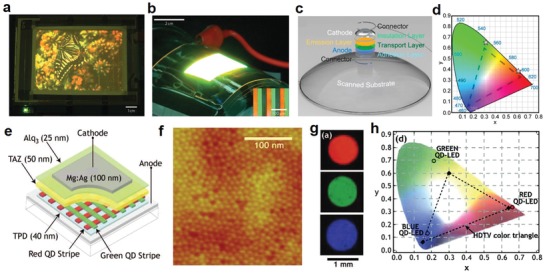
LEDs from quantum dots. a) Electroluminescence image of a full‐color QD display. b) Flexible LED with RGB QDs patterned by transfer printing (Inset: Magnified image of emission layer rows). Reproduced with permission.^196^ Copyright 2011, Springer Nature. c) 3D printed QD‐LED on a 3D scanned onto a curvilinear substrate. d) Color coordinates of the green (0.323, 0.652) and orange‐red (0.612, 0.383) QD‐LEDs marked by stars on the Commission International de l′Eclairage (CIE) 1931 chromaticity diagram, with dashed lines representing the HD television color standard saturation windows as defined by the National Television System Committee. Reproduced with permission.^41^ Copyright 2014, American Chemical Society. e) Schematic diagram shows the structure and materials of an archetypical QD‐LED. f) A high‐resolution AFM micrograph of a close‐packed monolayer of QDs deposited on top of the CBP hole transporting layer. g) Electroluminescent red, green, and blue QD‐LED pixels with the device structure shown in (e). h) Chromaticity diagram shows the positions of red, green, and blue QD‐LED colors, an HDTV color triangle is shown for comparison. Reproduced with permission.^154^ Copyright 2008, American Chemical Society.

Although QD‐LEDs have been integrated into electroemissive QD‐LED displays in laboratory settings (**Figure** **15**a,b), the device efficiency and lifetime are not satisfactory and need to be further improved for future commercialization.^149^ In pursuit of excellent cost‐effectiveness, further research is also ongoing in fully inkjet‐printed or gravure‐printed QLED displays.^151^ Two primary methods exist for the excitation of QDs for light emission that determines the structure of the devices. The first one is optical excitation, which relies on the use of external light to trigger a response in QDs, and has not seen large‐scale applications in printed diodes.^152^ The second one is direct electrical excitation, based on the application of current, resulting in QD‐LEDs with an emission layer sandwiched by electron transport and hole transport layers.^153^

The small size of QDs typically allows for relatively easy dispersion within solvents for ink preparation and printing. For example, McAlpine and co‐workers developed a 3D printing procedure to fabricate printed QD‐LEDs on a curvilinear substrate (Figure 15c).^41^ By interweaving five different materials to make up organic polymer charge transport layers, i.e., a QD emissive layer, an elastomeric matrix, solid and liquid metal leads, as well as a UV‐adhesive transparent substrate layer (Figure 15c), they demonstrated that the printed QD‐LEDs can generate highly saturated color emissions, enabling the creation of displays that can subtend the color gamut at levels greater than the high definition television standard (Figure 15d). Unfortunately, the lack of scalability of 3D printing techniques is blocking this method from industrial adoption, though it does form a basis for multilayer device fabrication via 3D printing in the future.

In another work done by Bulovic and co‐workers, a solvent‐free contact printing process for deposition of colloidal QD thin films as the electroluminescent layers within QD‐LEDs was proposed (Figure 15e,f).^154^ As shown in Figure 15g, red (CdSe/ZnS core–shell15), green (ZnSe/CdSe/ZnS core–double shell16,17), and blue (CdS/ZnS core–shell18) QD‐LED pixels were contact printed layer by layer to form identical structures. It was found that when the wide bandgap organic semiconductor, 4,4′‐N,N′‐dicarbazole‐biphenyl (CBP), replaced N,N′‐diphenyl‐N,N′‐di(m‐tolyl)benzidine (TPD) as a hole transport material, more efficient charge and exciton confinement, as well as an improvement in color saturation of the QD‐LEDs could be achieved (Figure 15h). The solvent‐free deposition of the QD monolayers is compatible with a wide variety of organic semiconductors that are not compatible with solution processing methods, and thus provides a new flexibility in choosing organic materials for improved device performance.

### 4.4 Organic Materials

Organic materials possess the highest scalability of any semiconducting material seen thus far, as well as the highest compatibility with solution processing and printing.^8^ The raw materials can typically be synthesized from solution on a large scale without high processing temperatures, enabling development of high‐throughput, cost effective fabrication through established printing techniques. Different from the fragility of inorganic materials commonly encountered after printing and thermal annealing, most organic materials are capable of retaining a superb degree of flexibility which renders them perfectly suited for flexible device applications.^3^ Moreover, organic polymers allow a quick modification of their chemical structure, electronic bandgap, ink solution properties, as well as mechanical properties through chemical synthesis in accordance to the necessary device requirements.^3^, ^155^

In comparison to inorganic materials, however, organic semiconductor materials possess some disadvantages in electrical performance due to their different generating mechanism of semiconducting properties.^155^, ^156^, ^157^ Within organic materials, conjugated π bonds of alternating single and double bonds exist between the carbon atoms, generating the delocalization of electronic charge in the relevant areas of the molecules.^5^, ^156^, ^158^ As a result, electrons in organic materials may demonstrate carrier mobility by hopping between localized states of molecules or thermally assisted tunneling methods, which typically result in lower mobility than that in the inorganic semiconductors with band transport mechanism. The weaker intermolecular bonds in organic materials have also led to inferior mechanical and thermodynamic properties and higher deterioration rates compared to those of inorganic materials.^155^ Based on the basic transport mechanism of organic semiconductors, it is believed that the most important parameters affecting charge carrier mobility in fabricated devices include the degree of crystallinity in organic layers, the degree of precision in control over the molecular structure, and the quality of chain alignment in layer deposition.^1^, ^159^ Although organic materials may be lacking in durability and lifespan, they are known for high flexibility, impressive uniformity, stretchability, and low weight, all of which are highly coveted features in modern flexible electronics applications such as RFID tags.^1^

#### 4.4.1 Small Molecules

**Figure 16:**
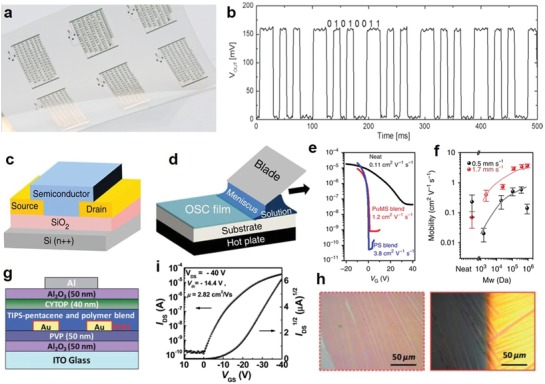
Electronics applications of the organic small molecules. a) Photograph of 8 bit RFID transponder chips on a plastic foil, based on a blend of TIPS‐pentacene and the polymer polystyrene. Each transponder has a footprint of 34 mm^2^. b) Output oscillations for an 8‐bit RFID transponder, with ink‐jet printed TIPS‐PEN:PS blend as the active layer. Reproduced with permission.^163^ Copyright 2013, Elsevier. c) BCBG device architecture and d) blade‐coating set‐up. e) Transfer characteristics of OTFTs prepared using neat diF‐TES‐ADT, diF‐TES‐ADT:PαMS (100 kDa) and diF‐TES‐ADT:PS (123 kDa) blends at a blade speed of 1.5 mm s^−1^ and a stage temperature of 70 °C. We employed *V*_ds_ = −10 V for blends and *V*_ds_ = −20 V for neat OSC. f) Hole mobility of OTFTs fabricated by blade‐coating diF‐TES‐ADT:PS blends using different Mw of PS both in different blade speeds. Reproduced with permission.^165^ Copyright 2015, Springer Nature. g) Schematic illustration of OFETs with the dual gate geometry made of TIPS‐pentacene/polymer blend film. h) TIPS‐pentacene/polymer blend films morphologies of PTAA blend layers processed from tetralin. i) Transfer characteristics of TIPS‐pentacene/poly‐(triarylamine) (PTAA) blend OFETs with top gate geometry processed from tetralin; CYTOP (700 nm) single layer gate dielectric. Reproduced with permission.^166^ Copyright 2012, Royal Society of Chemistry.

In many cases, organic materials can be easily solution‐processed and prepared as inks for use in printing. However, some difficulties may appear in the solution‐processing of organic materials in the small molecule category.^1^, ^160^ These materials are typically deposited through evaporation alongside the use of shadow masks, but efforts toward solution‐processing for higher scalability are in progress. In this regard, like most inorganic materials, a precursor route can be used.^161^ For instance, the functionalization approach used for CNTs can also be applied to organic materials, in which solubilized side chains are mixed with the small molecules for chemical attachment to prevent clumping and promote solubility.^162^ Kjellander et al. applied this approach with the inkjet printing of 6,13‐bis(triisopropyl‐silylethynyl)pentacene and polystyrene as the active layer of transistors, displaying a saturation mobility of 0.5 cm^2^ V^−1^ s^−1^.^163^ They further successfully built inverters, NANDs, and oscillators, then integrated nearly 300 TFTs in a matrix possessing the surface area of 34 mm^2^ for 8 bit RFID transponder circuits (**Figure** **16**a,b). However, due to the limitations of inkjet printing in controlling the morphology of 6,13‐bis(triisopropylsilylethynyl) (TIPS)‐PEN crystals, a large variation of performance in transistors was observed.^163^

Another useful solution‐processing approach is the blending of the molecules with a solution of polymers, which acts as a binder to form a framework for higher stability.^68^ In many cases, this strategy can assist in substrate compatibility and film formation, ensuring the deposition of an even active layer that is able to stably bind to the substrate.^164^ For instance, Niazi et al. splendidly proved the efficacy of this procedure with a blend of the 2,8‐difluoro‐5,11‐bis(triethylsilylethynyl)anthradithiophene (diF‐TES‐ADT) organic small molecule with the amorphous insulating polymer polystyrene/poly(α‐methylstyrene) (PS/PαMS) which was deposited via blade coating (Figure 16c,d).^165^ As shown in Figure 16e,f, the blended mixture of polymer and small molecule did give a higher hole mobility and on–off current ratio, but also smaller threshold voltage. The best TFT device fabricated with this approach reached a carrier mobility as high as 6.7 cm^2^ V^−1^ s^−1^.^165^ Another work by Hwang et al. used a mixture of TIPS–pentacene, poly (α‐methyl styrene), and poly (triarylamine) polymer matrices, along with tetralin as a solvent to fabricate solution‐processed organic field‐effect transistors (OFETs) of the structure in Figure 16g,h with mobility values up to 2.82 cm^2^ V^−1^ s^−1^.^166^ As the methods have been demonstrated to be capable of consistently generating well‐performing TFTs of low variability, the overall strategy can be considered potentially suitable for high‐throughput manufacturing on a larger scale.

**Figure 17:**
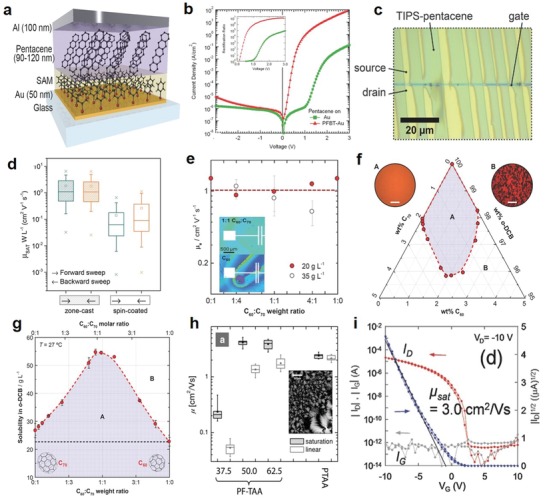
Electronics made of pentacene and C_60_. a) Structural schematic of a pentacene diode with self‐assembled monolayers of 2,3,4,5,6‐pentafluorobenzenethiol (PFBT) coated on the Au anode. b) The current density response of the structure in (a) to various biasing voltages (Inset: Corresponding diode rectification ratios). Reproduced with permission.^168^ Copyright 2016, Wiley. c) Optical micrograph image of zone‐cast TIPS‐pentacene crystals on a self‐aligned structure. d) Recorded mobility for zone‐cast and spin‐coated TFT devices with the structure of (c). Reproduced with permission.^169^ Copyright 2015, Wiley. e) Saturation mobility of C_60_:C_70_ films as a function of the weight ratio (Inset: Images of C_60_:C_70_ film (above, full coverage granted) and C_60_ film (below, incomplete coverage)). f) Ternary phase diagram for C_60_:C_70_:O‐DCB with a blue‐shaded single‐phase region and a two‐phase region corresponding to transmission optical images seen to the top left and right, respectively. g) Diagram of C_60_:C_70_ solubility as a function of the weight ratio. Reproduced with permission.^171^ Copyright 2015, Wiley. h) Saturation and linear carrier mobilities of diF‐TES‐ADT:PTAA and diF‐TES‐ADT:PF‐TAA blend OTFTs. Reproduced with permission.^172^ Copyright 2012, Wiley. i) Transfer characteristics of a C_10_‐DNBDT transistor. Reproduced with permission.^173^ Copyright 2015, Wiley.

Pentacene is a polyacene analogue which has distinguished itself as the primary organic material candidate for high‐performance HF and UHF operation due to its high charge carrier mobilities.^167^ Nonsolution processed pentacene‐based diodes have reached into the HF range, even touching the UHF range, while solution‐processed printed pentacene‐based diodes can at most be expected to remain in the 13.56 MHz band in the HF range. Up to now, the highest‐performing organic diode was built for a 1.24 GHz rectifier, which included a pentacene‐based diode with Au and Al contact electrodes (**Figure** **17**a).^168^ The pentacene active layer was deposited through thermal evaporation on a shadow mask with a glass substrate, while the Au electrode was given treatment with self‐assembled monolayers to ensure efficient charge injection. While possessing a 3 dB frequency at 1.24 GHz, the peak output voltage of 3.8 V was reached at a 1 GHz AC input frequency (Figure 17b), fully demonstrating the strong potential of pentacene‐based diodes for future UHF applications. Prominently, Higgins et al. produced state‐of‐the‐art solution‐processed OFETs based on TIPS–pentacene deposited through zone‐casting (Figure 17c), a large‐area compatible deposition method and nanoimprint lithography was used for patterning the device, allowing channel lengths down to 375 nm.^169^ Frequencies in the lower end of the HF band ranging from 1 to 9.8 MHz depending on the biasing voltage were observed, along with the mobility values shown in Figure 17d.

Another prominent semiconducting small organic molecule is buckminsterfullerene (C_60_) which has distinguished itself through higher mobility values.^170^ de Zerio Mendaza et al. demonstrated TFTs formed from a blend of C_60_ and 9% polystyrene of high molecular weight which possessed mobility up to 1 cm^2^ V^−1^ s^−1^ on a SiO_2_ substrate (Figure 17e–g).^171^ Other work by Smith et al. employed a blend of the diF‐TES‐ADT small organic molecule with poly(dimethyl‐triarylamine) (PTAA) and poly(dialkyl‐fluorene‐co‐dimethyl‐triarylamine) (PF‐TAA) as amorphous polymeric semiconducting binders to form polymer matrices.^172^ A significant enhancement of hole transport was observed, leading to field effect mobilities above 5 cm^2^ V^−1^ s^−1^ in the fabricated top‐gate OFETs, which can be considered encouraging for the future of solution‐processed organic small molecules (Figure 17h). Uno et al. incorporated decyldinaphthobenzodithiophene (C_10_‐DNBDT) within TFTs possessing mobilities reaching 16 cm^2^ V^−1^ s^−1^ through an “edge‐casting” fabrication technique, in which a crystalline domain is grown along the direction of an inclined substrate from a solution droplet sustained on an edge (Figure 17i).^173^ The utilization of these TFTs within half‐wave rectifiers yielded impressive operation frequencies up to 22 MHz with a 2 µm channel length, and later integration of the rectifiers with a 13.56 MHz transponder successfully powered a 5‐stage ring oscillator.

#### 4.4.2 Polymers

**Figure 18:**
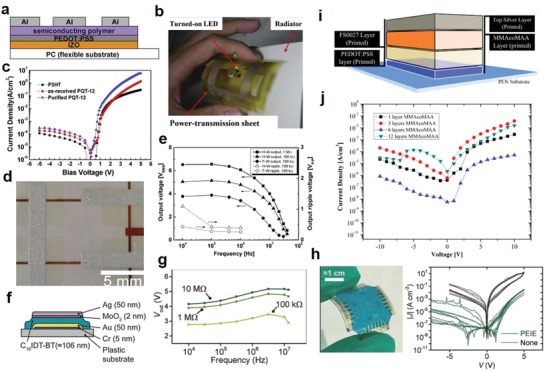
Schottky diode from organic polymers. a) Schottky diode structure including a PEDOT:PSS hole injection layer. b) Fully functional flexible wireless power transmission sheet with PQT‐12‐based Schottky diodes fabricated via spin‐coating with the structure of (a). c) Current density as a function of bias voltage for the diodes with three polymer varieties. Reproduced with permission.^175^ Copyright 2011, Elsevier. d) Photograph of gravure printed full‐wave circuit including four PTAA‐based Schottky diodes. e) Output voltage as a function of operation frequency for various rectifier setups with the diodes of (d). Reproduced with permission.^11^ Copyright 2013, IEEE. f) C16IDT‐BT‐based diode structure. g) Output voltage as a function of frequency for half‐wave rectifiers with spin‐coated Schottky diodes based on the design of (f). h) Image of the diode on a flexible substrate and the *I*–*V* characteristics for the diode both with and without a PEIE interlayer separating the cathode and semiconductor. Reproduced with permission.^177^ Copyright 2017, Wiley. i) Fully inkjet‐printed PTAA‐based MIS diode architecture. j) Plot of current density as a function of voltage and the number of MMAcoMAA layers for the device structure in (i). Reproduced with permission.^178^ Copyright 2017, IOP Publishing.

In comparison to small molecules, polymers have seen far more research in the fabrication of solution‐processed semiconductor devices capable of RF operation. An early example of gravure printing was presented by Lilja et al., who used roll‐to‐roll gravure printing to form rectifying organic Schottky diodes on a metallized polyester (PET) film.^174^ PTAA was used as the active layer, with a silver layer as the anode and a patterned copper layer as the cathode. After incorporation within a rectifier, the operation frequency reached about 10 MHz with an input AC voltage 10 V in amplitude, alongside a rectification ratio over 10^4^. Later, Lin et al. employed the lower‐throughput spin coating approach to form vertical Schottky diodes employed in rectifiers which did reach the 13.56 MHz operation band, thus qualifying for standard HF operation.^175^ As presented in **Figure** **18**a, they utilized poly(3,30 00didodecylquaterthiophene) (PQT‐12) as the active layer, along with indium zinc oxide (IZO) and Al as the anode and cathode electrodes, respectively. After incorporation into a rectifier with a function generator input, an output‐smoothing capacitor, an oscilloscope probe, and a 1 MΩ load resistance, the resulting rectification ratio reached as high as 2 × 10^4^ with a forward current density of 6 A cm^−2^ while assuming an AC input voltage of 5 V in amplitude (Figure 18b,c). The operation frequency reached up to 14 MHz.

Building on previous work, Heljo et al. progressed even further, again fabricating 13.56 MHz organic Schottky diodes through gravure printing.^11^ PTAA was used as the active layer with Cu and Ag electrode layers, and incorporation into two rectifier configurations with full wave and half wave circuits both resulted in functional 13.56 MHz operation (Figure 18d,e). The maximum output voltage achieved reached 3.5 V at 13.56 MHz with an AC input voltage of 10 V in amplitude, and the fabrication process itself demonstrated excellent reliability at a 97% device yield.^176^ Higgins et al. later successfully took device performance up another notch, again at the cost of throughput, employing spin coating with an indacenodithiophene‐benzothiadiazole copolymer (C_16_‐IDT‐BT) in a Schottky diode using Au and molybdenum trioxide/silver (MoO_3_/Ag) electrodes as the cathode and anode, respectively (Figure 18f–h).^177^ A high reverse breakdown voltage nearly reaching 15 V was observed, as well as a rectification ratio exceeding 10^6^ with input voltages between −5 and 5 V.

As an alternative to gravure printing, Mitra et al. demonstrated fully inkjet‐printed metal‐insulator‐semiconductor (MIS) diodes with a PTAA active layer, Ag electrodes, and a poly (methylmethacrylate‐methacrylic acid) (MMAcoMAA) insulating layer,^178^ as seen in Figure 18i. Given 10 V amplitude AC input voltages, the maximum forward current density was measured to be 40 mA cm^−2^ after layer structure optimization as indicated in Figure 18j, corresponding to rectification ratios in the range of 10^3^–10^4^. These results suggest that organic polymers are nearly certain to enter the commercial sector of RF semiconducting devices operating within the HF band in the future. The results achieved by groups utilizing organic semiconductors in nonphotonic applications are included in Table 1.

#### 4.4.3 Optoelectronics Applications

In recent years, great interest has been focused on the development of organic photodiodes for existing imaging technologies due to the combination of their lightness of weight, flexibility, biocompatibility, tunable photophysical or optoelectronic properties, and compatibility with both large and small areas.^179^ Recent research has mainly concentrated on the tuning of solution‐processable organic semiconducting materials, fabrication procedures, and device architectures for the optimization of performance parameters. For example, Eckstein et al. recently demonstrated fully printed, flexible, and semi‐transparent organic photodiodes with an aluminum‐doped zinc oxide (AZO) nanoparticle hole‐blocking layer, a blended polythieno[3,4‐b]‐thiophene‐co‐benzodithiophene (PTB7) and [6,6]‐phenyl C71‐butyric acid methyl ester (PC70BM) bulk heterojunction active layer, and PEDOT:PSS electrodes for a PEDOT:PSS (200 nm)/AZO NP (50 nm)/PTB7:PC70BM (310 nm)/PEDOT:PSS (200 nm) structure.^180^ The all‐printed organic photodiode possessed an external quantum efficiency (EQE) of 50%, device transparency of ≈15%, a specific detectivity (D*) above 10^11^, a spectral response of 0.26 A W^−1^, and a ≈300 kHz band width at −3 V reverse bias, exceeding nearly all comparable devices of its category. The performance of organic photodiodes has caught up to, and even exceeded that of inorganic photodiodes in multiple areas. Higher EQE values up to 80% have been observed in fully inkjet‐printed organic photodetectors using a poly(3‐hexyltiophene) and [6,6]‐phenyl‐C 61 butyric acid methyl ester bulk heterojunction photoactive layer with an Ag bottom electrode and PEDOT:PSS top electrode built on a PEN substrate.^181^ Combined with the inexpensive and scalable nature of printing, the future integration of organic photodiodes with suitable applications in large area flexible digital imagers, bioelectronics, surveillance systems, and other fields is rapidly approaching realization.^179^, ^182^

In the OLED field, Dongwon et al. developed full‐color polymer LED displays deposited through inkjet printing after careful optimization of ink formation and drying procedures for uniform film profiles and uniform photoemission.^183^ The need for complex interlacing techniques was reduced with an additional hole‐conduction layer for charge transport. More recently, Kopola et al. reported the fabrication of OLED structures with modified PEDOT:PSS layers for lighting applications through gravure printing on glass substrates, reaching luminosities up to 1000 cd m^−2^ with a 5.4 V input voltage.^184^ They demonstrated large‐area fabrication up to 30 cm^2^ with fully gravure printing of the polymer layers, further reinforcing the feasibility of OLED production via printing.^185^

Research regarding the printed solar cells has also steadily leaned toward the use of organic materials due to their processability in low temperatures, compatibility with flexible substrates, and relatively lower cost in photovoltaic applications.^186^, ^187^ In performance, the most efficient organic solar cells (OSCs) have demonstrated power conversion efficiencies in the range of 10–13% in single junction cells owing to the employment of nonfullerene acceptor and conjugated polymer donor materials in combination with a buffer layer for separating the active layer from the electrodes to increase device efficiency.^188^, ^189^ In one notable example, by modifying the molecular structure of the low bandgap n‐type organic semiconductor 3,9‐bis(2‐methylene‐(3‐(1,1‐dicyanomethylene)‐indanone))‐5,5,11,11‐tetrakis(4‐hexylphenyl)‐dithieno[2,3‐d:2′,3′‐d′]‐s‐indaceno[1,2‐b:5,6‐b′]dithiophene (ITIC) via side‐chain isomerization with meta‐alkyl‐phenyl substitution, Yang et al. developed spin‐coated polymer solar cells (PSCs) that could reach power conversion efficiency of 11.77%, one of the highest reported efficiency values in nonfullerene PSCs.^190^ Despite great strides in performance, printed organic solar cells are still far from competing with the state‐of‐the‐art single‐junction tandem solar cells which have already shown a power conversion efficiency over 27.3%.^186^, ^188^

## 5 Conclusions

In this review, we have systematically examined the unique materials, printing technologies, and different kinds of devices and applications of printed diodes. In all the materials used for diode applications, organics are undoubtedly the most promising candidates for printing methodologies due to their solution‐processing compatibility and lower cost. However, organic diodes will likely remain locked in the HF applications in the foreseeable future due to their inherent drawbacks. The current fabrication for printed organic diodes is close to maturity, and further development may remain reliant on the introduction of new materials such as the high‐mobility soluble small molecules or novel polymer/small molecule blends. By combining the high uniformity of organic materials suited for large‐area deposition and the high‐throughput traits of printing, printed organic diodes can be expected to boast the least expensive prices of all material classes in the future. In the field of photonics, printed OLED‐based device applications in the textile, lighting, display sectors are unlikely to outperform peers in lifespan or overall performance, but possess a significant chance of becoming competitive within the next decade of development by virtue of far more accessible cost.

After organics, silicon may be another good material for printed semiconducting devices. While current focus is on the transfer of prefabricated Si nanomembranes to flexible substrates, this method has only yielded excellent results on a small scale and faces severe issues in throughput and cost‐effectiveness. Depending on development, solution processing could become a scalable fabrication process while remaining suitable for applications in the UHF and perhaps even microwave bands. Although MOS such as IGZO showed excellent performance in HF, UHF, and even higher applications in rectifiers and additional circuitry with the vacuum deposition method, solution processed fabrication is still facing difficulties in the form of the high annealing temperatures needed for MOS precursor solutions, especially in the case of IGZO. However, the possibility does exist that binary or more complex oxides may yield a new material able to provide an easier route to solution‐processed ink for printing.

The unique electrical and mechanical properties of nanomaterials, such as excellent durability, unmatchable flexibility, and high charge carrier mobilities as well as solution‐processing compatibility, render them exceptionally suitable for flexible semiconducting applications and may represent the greatest hope of printing diodes capable of microwave‐band operation, competing with silicon. Nevertheless, their unique properties have also led to frustrating fabrication challenges, such as the impedance mismatch, alignment, and metallic impurity issues seen in CNTs. While printed diodes are nearly certain to enter large‐scale production for device applications in the HF band employing organic materials as well as in photonics applications, their level of involvement in UHF and microwave bands will greatly depend on the degree of success achieved in research involving nanomaterials and inorganic materials.

Furthermore, in printing fabrication, to achieve high performance, it is critically important to carefully tune the ink properties, printing procedure type, and substrate surface during deposition as well as later annealing or other treatment steps to dry or remove impurities from the deposited ink. The materials used tend to suffer from fabrication issues of a severity degree inversely proportional to their degree of potential, with carbon nanomaterials being associated with the most difficulties and organic polymers the least. In addition, the need to achieve good metal–semiconductor interfaces is still a major challenge using printing technologies, and low‐temperature processes are critically needed. Taking into consideration the significant commercial potential of printed diodes and their suitability for flexible applications, strong interest will only continue to rise in the coming future.

## Conflict of Interest

The authors declare no conflict of interest.
